# From rest to task: temporal mechanisms of self-association

**DOI:** 10.1098/rstb.2025.0346

**Published:** 2026-11-10

**Authors:** Georg Northoff, Xinyu Shen, Andrea Avramescu, Jie Sui

**Affiliations:** ^1^ Institute of Mental Health Research, University of Ottawa, Ottawa, Canada; ^2^ Institute of Psychology, Chinese Academy of Sciences, Beijing, People's Republic of China; ^3^ University of Aberdeen, Aberdeen, Scotland, UK

**Keywords:** self-referential processing, temporal matching, temporal integration, temporal compression, intrinsic self-representation, rest–task interactions

## Abstract

Our self is characterized by both ongoing, long-lasting intrinsic self-representation in spontaneous brain activity and short-term, transient self-association with extrinsic stimuli during task-related activity. However, how these two dimensions of the self are related remains unclear. How do they interact when extrinsic stimuli become associated with one's self-representation? Addressing this gap in our knowledge is the goal of our paper. We propose three temporal mechanisms that may connect spontaneous and task-related activity during sequential self-processing stages: (i) temporal matching of spontaneous activity's intrinsic self-representation with extrinsic stimuli through the brain's scale-free dynamics; (ii) temporal integration of the shorter extrinsic stimulus duration within the longer temporal windows of the spontaneous, intrinsic self-representation using the brain's intrinsic neural timescales; and (iii) temporal differentiation by which the self-specificity of extrinsic stimuli is distinguished from intrinsic self-representation via transient suppression and compression of ongoing spontaneous activity. Together, these three temporal mechanisms may shed light on the nature of self–stimulus associations by explicating the interaction between intrinsic self-representation and task-related extrinsic stimulus processing. This novel temporal framework extends current theories by proposing a hierarchical temporal model of self embedded within a dynamic layer model of the brain. This framework may have major implications for an artificial self in current artificial intelligence technologies.

This article is part of the discussion meeting issue ‘From being to identity: analysis and synthesis of the self’.

## 1. Introduction

The self is at the core of our mental experience. Our sense of self persists over time despite continuous changes to both body and mind, suggesting a temporally stable and *intrinsic self-representation* [1–3]. Moreover, we perceive extrinsic events and objects in relation to the self, performing a *self-association with extrinsic stimuli* or an *extrinsic self–stimulus association* [1,2,4]. This phenomenon has been referred to in various ways, such as self-relatedness, self-reference, self-specificity, or self-prioritization [1,2]; for the sake of simplicity, we use these terms synonymously. However, we still do not know *how* these two dimensions, intrinsic self-representation and self-association with extrinsic stimuli, are related. Addressing this gap in their relationship is the focus of our paper.

How can our self manifest both as a persistent intrinsic self-representation and as a transient stimulus-evoked representation during self-association with extrinsic stimuli? This suggests a fundamentally temporal organization of the self through which persistent and transient features are integrated across neural and psychological levels [5–9]. Therefore, we propose a temporal model of the self. According to this model, the temporal organization of the self may unify its different manifestations across several domains, including bodily and minimal selfhood [10–20], mental and autobiographical selfhood [13,21–26], social and emotional selfhood [13,22,27–33], and core dimensions of self-experience such as self-identity [34–36], self-consciousness [7], agency, and ownership [16]. For further details on these domains and dimensions of the self, see boxes 1 and 2. Here, we focus on three temporal mechanisms that may characterize the self's temporal organization and link intrinsic self-representation with extrinsically triggered self-association: temporal matching, temporal integration, and temporal differentiation.

**Box 1. Temporal mechanisms and the predictive coding model of self**

The predictive coding model of self (PCMS) [10,12,23,37–44] is a prominent theory which postulates that on a stochastic basis, the brain generates models that allow it to actively infer the hidden causes behind the inputs it receives [45]. In this model, predictions function as empirical priors that shape the processing of subsequent inputs, whereas mismatches between prediction and input generate prediction errors. Predictive coding has been especially influential in accounts of the bodily self, where self-related experience is understood as arising from the integration of interoceptive, proprioceptive, and exteroceptive signals under predictive constraints [44].

Our three temporal mechanisms are compatible with this predictive coding account but specify it in temporal-dynamic terms. Temporal matching converges most directly with predictive coding because it concerns the relationship between intrinsic neural activity and extrinsic stimulus structure. If spontaneous activity contributes to prediction, as recent work suggests [46,47], then stronger scale-free dynamics in spontaneous activity, indexed by higher power law exponent (PLE), may reflect a stronger intrinsic temporal prior for subsequent self-association with extrinsic stimuli. In this sense, temporal matching may provide a dynamic mechanism through which intrinsic neural stochastics shape the processing of extrinsic natural stochastics. Whereas predictive coding describes this process computationally in terms of priors and predictions, temporal matching specifies how these priors may be instantiated in the temporal structure of spontaneous activity.

Temporal mechanisms also complement the basis model of self-specificity (BMSS) [1] and the self-attention model (SAM) [2,48]. The BMSS assigns a central role to spontaneous activity in self-specific processing but leaves open the precise functional mechanism by which spontaneous activity contributes to self-association. Temporal matching addresses this gap by suggesting that spontaneous activity provides temporal priors that constrain the association of extrinsic stimuli with the self. These temporal priors may then facilitate the attentional enhancement of self-related stimuli proposed by the SAM. Thus, the BMSS and SAM can be linked through the temporal-dynamic role of spontaneous activity: spontaneous activity provides intrinsic temporal priors, and these priors may bias subsequent attention towards self-associated stimuli.

Finally, temporal differentiation may also be interpreted within a predictive coding framework. In dynamic terms, we describe pre-stimulus variability, entropy, and complexity as features of ongoing spontaneous activity that shape post-stimulus processing. In predictive coding terms, these pre-stimulus features may correspond to the flexibility or precision of empirical priors before stimulus onset. Post-stimulus reductions in trial-to-trial variability (TTV), entropy, and Lempel–Ziv complexity (LZC) may then reflect the dynamic consequences of stimulus processing, including the reduction, resolution, or expression of prediction error. In this view, higher pre-stimulus variability or complexity may indicate a more flexible prior that can be more strongly shaped by the extrinsic stimulus, leading to lower post-stimulus variability, entropy, or complexity during self-specific processing [49]. This proposed mapping between dynamic measures and predictive coding constructs remains speculative, but it generates a testable dynamic-computational hypothesis: pre-stimulus dynamics should predict post-stimulus signal compression and prediction error-related processing during self-association.

**Box 2. Temporal mechanisms, the minimal-narrative self, and the dynamic pattern theory of self**

While there are many conceptual definitions of the self, an important distinction to make is between the minimal self and the narrative self [50–53]. The minimal self, commonly referred to as the bodily self, consists of the basic experience of our sense of self and our body [54] and is intimately linked to somatic and affective bodily processes [55,56]. In this way, the minimal self mirrors other closely related concepts, such as the basic self [57], the core self [13], the pre-reflective self [33] and the experiential self [33,55]. By contrast, the narrative self [16] requires further cognitive elaboration and self-awareness using processes such as attention and working memory and is highly sensitive to context and environment [58]. Similar definitions of self include the autobiographical self [13], the empirical self [59], the cognitive self [60] and the objective self [61].

Thus, the minimal self and the narrative self can be understood as distinct but deeply interconnected dimensions of selfhood. We propose that the three temporal mechanisms, temporal matching, temporal integration, and temporal differentiation, operate across both minimal and narrative selfhood, as well as across other concepts of the self. What differs across these concepts is not the presence or absence of temporal organization but the relative weighting of shorter and longer timescales. The minimal self may be more strongly grounded in shorter timescales of bodily, interoceptive and sensorimotor processing, whereas narrative selfhood may involve longer timescales of autobiographical memory, self-identity and temporal continuity. Accordingly, timescales and temporal mechanisms may provide a way to connect and partially unify different concepts of the self without reducing them to a single content-based definition.

However, another concept of the self is the dynamic pattern model of self (DPS) [62,63]. The DPS describes the self not as a particular entity but as a structure or pattern of various dynamic processes, including social, ecological, somatic, and cognitive processes. In this way, the DPS may be specified by our three temporal mechanisms. Temporal matching enables the system to detect which inputs align with intrinsic temporal dynamics, creating the basis for self-relevance detection. Temporal integration binds these distributed processes across time, forming coherent patterns from moment-to-moment fluctuations. Finally, temporal differentiation abstracts the integrated patterns, enabling both stability (pattern persistence) and flexibility (pattern updating). Together, while the DPS characterizes what patterns emerge and their functional roles, our temporal model of self articulates how these patterns arise from specific temporal operations on neural dynamics, namely, through our three temporal mechanisms.

Research on the neural basis of the self has largely followed two major lines of inquiry: task-related activity and spontaneous activity. On the one hand, studies focusing on task-related activity have documented specific differences during the processing of extrinsic self-related stimuli, such as one's own name or face, compared with non-self-related stimuli. These findings suggest that extrinsic self-related stimuli elicit task-specific neural responses during self-association [2,3,48,64]. On the other hand, there is increasing evidence that the spontaneous resting-state activity of the brain is associated with one's sense of self [65,66], suggesting that spontaneous activity may contain an intrinsic self-representation prior to extrinsic self–stimulus association. The relevant findings are summarized in table 1 and figure 1—for additional details, see the supplementary material.

**Table 1.** Overview of neural studies on self-association during task-related activity (upper) and intrinsic self-representation in resting-state activity (lower). (The table summarizes existing neural evidence linking task-related activity to self-association with extrinsic stimuli (upper part) and spontaneous activity to intrinsic self-representation (lower part). Upper part: event-related potentials (ERPs), which reflect the mean amplitude averaged over time and trials, dominate studies of self-association with extrinsic stimuli during task-related activity. Lower part: dynamic measures, which capture patterns of change in amplitude and/or connectivity over time, dominate studies on the neural signature of intrinsic self-representation in the spontaneous activity of the brain. Further empirical details are provided in the supplementary material, and different concepts of the self are discussed in boxes 1 and 2.)

processing mode	neural marker	specific component	key evidence/observation	functional implication	key references
**task-related (post-stimulus)**	late ERPs	P3 (P300) (300-500 ms)	amplitude increases for self-specific stimuli (name, face, car, etc.) across domains. Modulates decision thresholds (hierarchical drift diffusion model)	indicates domain-independent self-prioritization; requires less evidence to reach a decision	Berlad & Pratt (1995) [67]; Sui *et al.* (2023) [68]; Tacikowski & Nowicka (2010) [69]
	early ERPs	N1/N2 (early latency)	decreased N170/N2 for self-faces (less surprise). Negative N1 amplitude stronger for self-matching; correlates with faster drift rates	reflects early automatic processing and successful association of self with stimulus; faster/more accurate responding	Bola *et al.* (2021) [70]; Sui *et al.* (2023) [68]; Pinheiro *et al.* (2023) [71]
	functional connectivity (FC)	alpha band (8–12 Hz)	frontal-to-occipital: increases 150–180 ms post-stimulus for self-matching. Occipito-frontal: weaker late feedforward FC for self-trials	suggests top-down feedback modulation (intrinsic self-projection) dominates over bottom-up sensory processing for self-stimuli	Lu *et al.* (2024) [72]; Kotlewska *et al.* [73]
	other bands	gamma, beta, delta	gamma (40 Hz) linked to self-reference; beta linked to motor/evidence accumulation	supports a global-temporal mechanism where multiple bands integrate to support self-cognition	Bocharov *et al.* (2019) [74]; Haciahmet *et al.* (2023) [75]
**spontaneous/** **intrinsic (resting and pre-stimulus)**	pre-stimulus activity	BOLD signal (functional magnetic resonance imaging (fMRI))	high activity in CMS (ventromedial prefrontal cortex, posterior cingulate cortex) before stimulus predicts higher self-specificity ratings	the brain's spontaneous activity contains baseline information about the self (the ‘prior’)	Schneider *et al.* (2008) [76]; Qin *et al.* [77]; Northoff *et al.* (2009) [78]
	pre-stimulus activity	alpha power (electroencephalography (EEG))	higher pre-stimulus alpha power predicts higher self-relatedness assignment	alpha may act as a gate, carrying self-information from pre- to post-stimulus states	Bai *et al.* [79]; Kraus *et al.* (2021) [80]
	temporal dynamics	power law exponent (PLE)	steeper spectral slope (high PLE) in infraslow (fMRI) and 1-50 Hz (EEG) correlates with high private self-consciousness	the balance of slow-to-fast frequencies encodes the intrinsic self-representation, specifically in the CMS	Huang *et al.* [81]; Wolff *et al.* [82]
	temporal dynamics	intrinsic neural timescales (INTs)	longer autocorrelation windows (ACW) correlate with higher private self-consciousness	the duration of neural processing windows mediates intrinsic self-representation	Wolff *et al.* [82,83]; Sugimura *et al.* [84]
	anatomy	cortical midline structures (CMS)	resting-state activity in default-mode network (DMN)/CMS overlaps spatially with task-related self-activity	provides the anatomical core for the intrinsic self-representation	D'Argembeau *et al.* (2005) [85]; Qin & Northoff (2011) [86]

**Figure 1.**
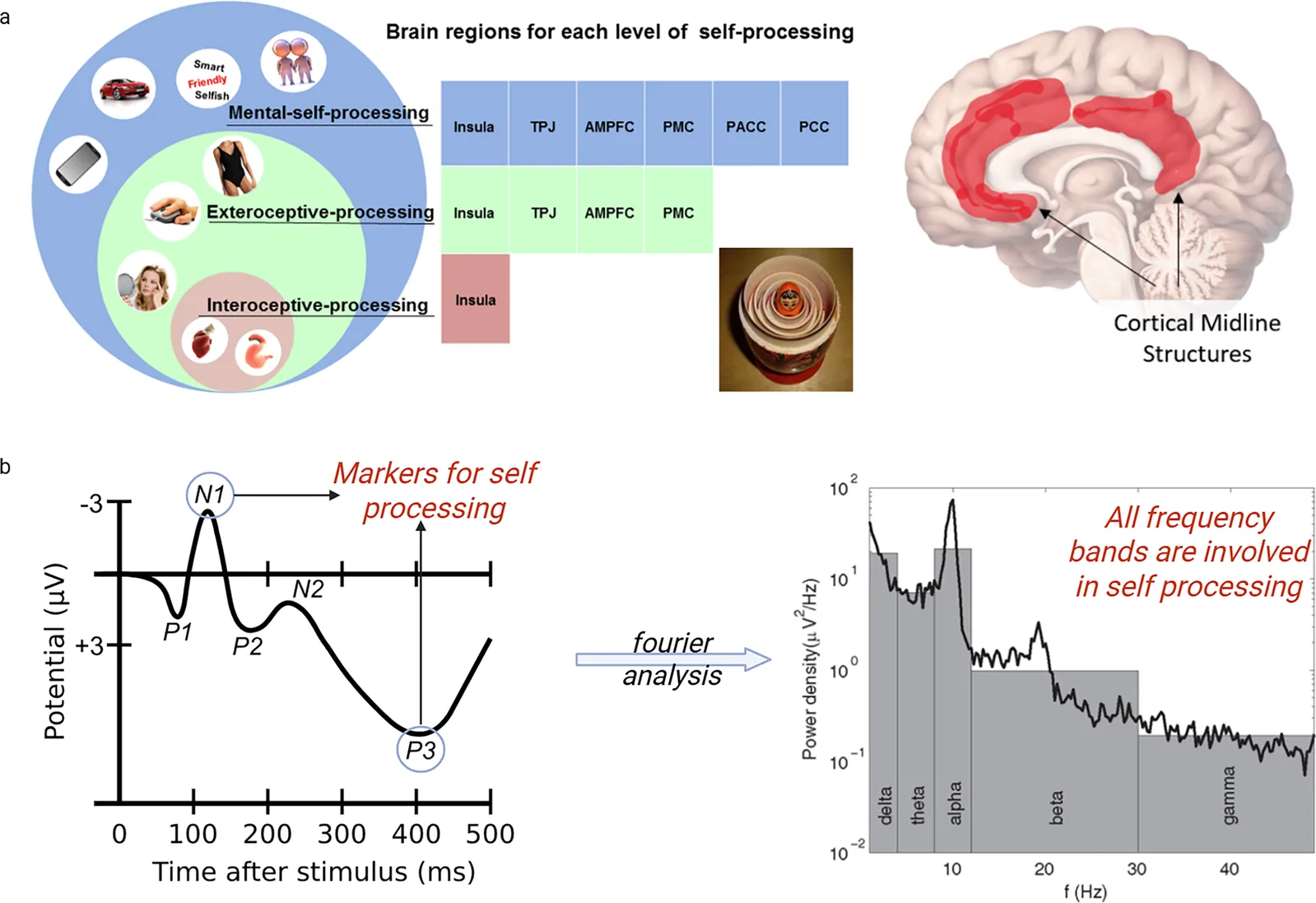
Neural basis of self-processing with spatial (a) and temporal (b) manifestations in the brain. (a) Spatial and hierarchical organization of self-processing: different domains of self-experience—ranging from interoceptive (bodily/minimal) to exteroceptive and mental self-processing—are nested hierarchically, functioning much like Russian dolls (left and middle part in panel (a). This intrinsic self-representation, which is already present during the resting state in the spontaneous activity of the brain, is supported by specific brain regions, most notably the insula and the cortical midline structures (CMS) (right part in panel (a)), as the core of the default-mode network. (b) Temporal and electrophysiological organization of self-processing: the temporal mechanisms of self-processing integrate both transient and persistent neural activity. Task-related activity during extrinsic self-stimulus association evokes specific transient electrophysiological changes, such as N1 and P3 event-related potentials (left part in panel (b)). Moreover, Fourier analysis demonstrated that the longer-lasting and more persistent spontaneous neural activity associated with intrinsic self-processing spans across all temporal frequency bands (delta through to gamma), signified by their different timescales (right part in panel (b)); this highlights a fundamental temporal organization that links the longer-lasting persistent resting-state's intrinsic self-representation (right) with the shorter, more transient task-related responses during self-association to extrinsic stimuli (left).

Moreover, extensive investigations of the spatial-topographic features of the self have revealed strong evidence implicating the default-mode network (DMN), particularly its cortical midline structures (CMS), in self-related activity across both resting and task states [13,22–26,50]. By contrast, the temporal features of the self have received much less attention. More importantly, despite some work addressing this issue at the neural [34,35,50] and psychological [51–53] levels, the relationship between intrinsic self-representation in the resting state and extrinsic self–stimulus association in task states remains unclear. Thus, our paper addresses this gap by proposing three temporal mechanisms that link resting-state intrinsic self-representation with task-related extrinsic self–stimulus association.

## 2. Proposed temporal mechanisms and dynamic measures of rest-task integration in self-representation

We propose three temporal mechanisms (figure 2) operating along distinct processing stages: temporal matching (§3), temporal integration (§4), and temporal differentiation (§5). These mechanisms link the intrinsic self-representation in spontaneous activity with self-association to extrinsic stimuli during task-related activity [65,66], thereby contributing to psychological ‘self-prioritization’ [2,3,48,64]. The three mechanisms are based on three core dynamic features of neural activity. By dynamic features, we refer to properties of neural activity that capture how brain signals continuously change across time and space [65]. These dynamic patterns can be measured in various ways from functional magnetic resonance imaging (fMRI) and electroencephalography (EEG)/magnetoencephalography (MEG) data by comparing relative signal changes from time point to time point across the full time series rather than averaging them into a mean.

**Figure 2.**
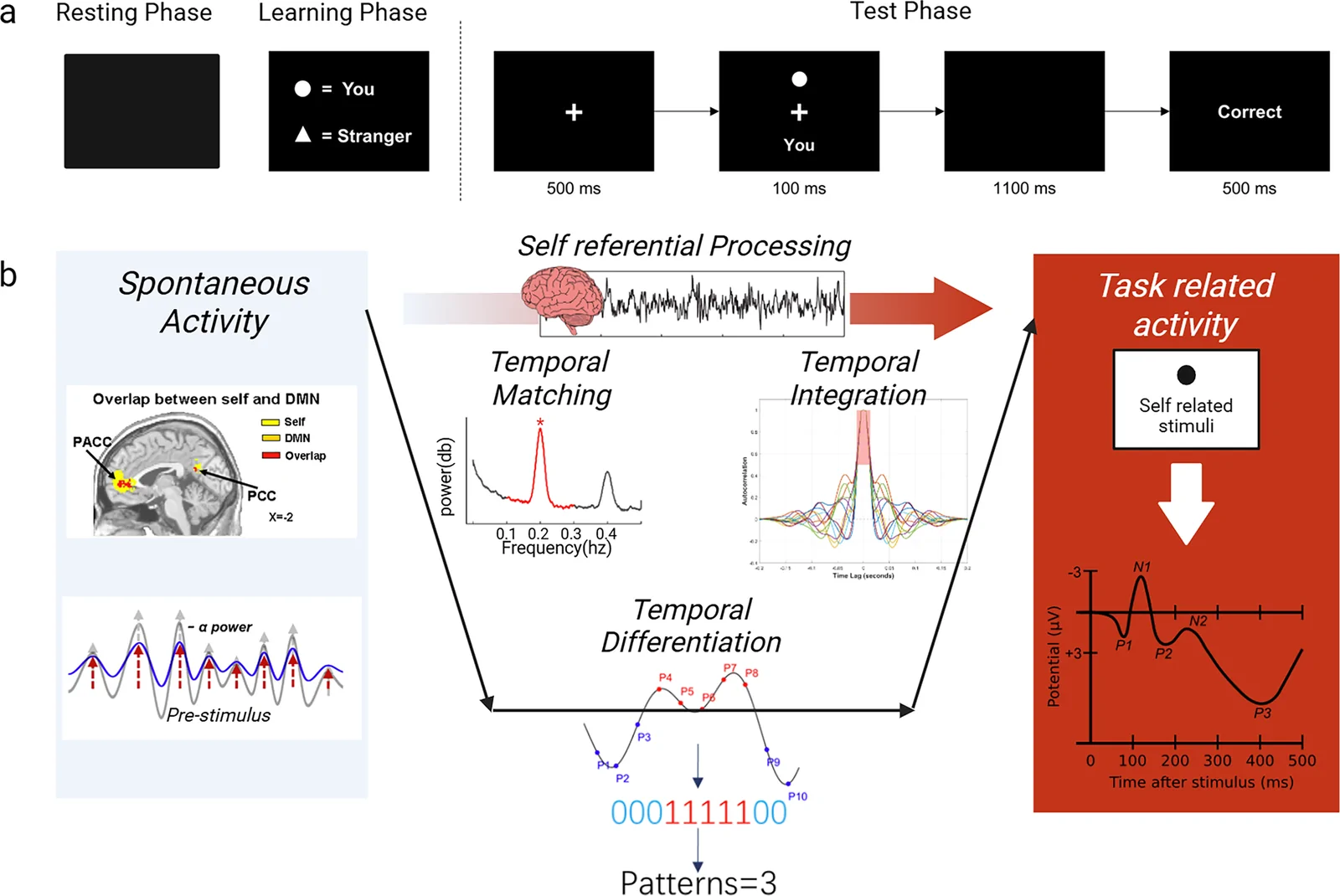
Overview of the three temporal mechanisms of self-association with extrinsic stimuli during the transition from spontaneous to task-related activity. (a) The experimental paradigm outlines the resting, learning, and test phases used to assess self-prioritization, wherein participants learn to associate extrinsic stimuli (e.g. shapes) with their own self, reflecting the intrinsic self-representation, or a stranger as a control for self-specificity. (b) Connecting the spontaneous activity of the brain and its intrinsic self-representation (left) with task-related activity for the self-association of extrinsic stimuli (right) through three temporal mechanisms (middle). Left panel: we show spontaneous brain activity and its intrinsic self-representation (upper left) as measured in resting-state cortical midline structures (CMS) (middle left) and pre-stimulus activity alpha power (lower left) before the task. Middle panel: the three temporal mechanisms, namely, matching, integration and differentiation, are shown; they provide the transition from spontaneous activity's intrinsic self-representation (left) to self-association to extrinsic stimuli (right). Each mechanism is measured and operationalized by a particular dynamic measure: scale-free dynamics (measured by the power law exponent (PLE)) for temporal matching, intrinsic neural timescales (INT; measured by the autocorrelation window (ACW)) for temporal integration, and information complexity (measured by Lempel Zev complexity (LZC)) for temporal differentiation; these are visualized below each mechanism in a schematic way. Right panel: this shows task-related activity during the self-prioritization task; here, the association of self with extrinsic stimuli occurs, e.g. self-association with extrinsic stimuli. Note that the temporal sequence of the experimental paradigm (a) matches the temporal sequence of the three temporal mechanisms (b).

The first dynamic feature is scale-free dynamics, quantified by the power law exponent (PLE), which captures the 1/f-like slope of the power spectrum and reflects the balance of power between slow and fast frequency fluctuations [81,87–92]. Scale-free dynamics describe a fractal-like organization of neural activity, where similar temporal patterns recur across faster and slower frequencies. In this structure, faster, lower-power fluctuations are nested within slower, higher-power fluctuations, a phenomenon referred to as temporal nestedness [87,93–95].

The second dynamic feature concerns the brain's intrinsic neural timescale (INT), which provides temporal windows for neural processing. The INT can be measured by temporal autocorrelation, which captures how strongly a signal remains related to itself over time. It is operationalized by the autocorrelation window (ACW), a measure of how long neural activity remains correlated across successive time points [83,96–101]. Stronger correlations across increasing temporal lags indicate a longer ACW, reflecting greater temporal continuity in neural activity [6,95,102]. Like the PLE, the ACW can be measured during both rest and task states [6,95,102]. However, another way of measuring INT during task states is the temporal receptive window (TRW), which measures how the duration or length of an extrinsic stimulus (such as words or sentences) relates to the duration of its neural processing in the brain [50,103].

The third dynamic feature concerns information processing in the neural signal, which can be assessed through measures of signal variability, complexity and entropy. One such measure is trial-to-trial variability (TTV), which captures post-stimulus reductions in amplitude variability across trials [82,89,104–106]. Another is Lempel–Ziv complexity (LZC) [107], which is associated with temporal signal compression and reflects the diversity of brain activity patterns when applied to neural data [94,108–110]. Finally, entropy measures the regularity-irregularity, predictability, order-disorder and information processing capacity (or repertoire) of the signal in terms of neural activity (see figure 1 and table 2 for details on the three dynamic features and their respective measures).

**Table 2.** Three proposed temporal mechanisms and their corresponding dynamic features and measures.

temporal mechanism	neurodynamic features and measure	domain: frequency/time domain	definition and computation	neural interpretation
**temporal matching**	*scale-free dynamics:* power law exponent (PLE)	frequency	computed by fitting the aperiodic 1/f slope of the power spectrum, usually on a log-log scale of power versus frequency. It quantifies the relative distribution of power across slow and fast neural fluctuations	the aperiodic exponent (PLE) is considered to reflect the overall excitability of brain networks and the excitatory-inhibitory (E/I) balance. Serving as a non-invasive proxy for E/I balance, aperiodic activity has been shown to play important functional roles in multiple cognitive domains
**temporal integration**	*intrinsic neural timescales* (INT): autocorrelation window (ACW)	time	computed from the autocorrelation function of a neural time series. It is defined as the lag at which the signal's autocorrelation decays to a specified threshold (e.g. 0.5 or 0), indexing the duration over which the signal remains dependent on its previous states	the brain operates within temporal receptive windows (TRW), integrating and segregating the information of extrinsic stimuli (like words or sentences) over specific periods of time. INT, which measure the temporal self-correlation of neural signals (ACW), are closely associated with TRW, reflecting an important relationship between neural temporal features and input/stimulus processing mechanisms
**temporal differentiation**	*information processing:* trial-to-trial variability (TTV)	time	computed as the variance or standard deviation of neural response amplitude across repeated trials at each time point during the post-stimulus period. It quantifies how consistently or variably the brain responds to the same stimulus across trials	TTV is not random background ‘noise’ or measurement error, but a vital reflection of rest-stimulus interaction, e.g. pre- and post-stimulus periods. Rather than a sensory input simply adding its signal onto a passive baseline, incoming information deeply interacts with the brain's highly structured, ongoing spontaneous activity. When a stimulus arrives, it typically triggers ‘variability quenching’—a temporary suppression of the brain's spontaneous fluctuations during the post-stimulus period that locks the cortex into a transiently stable, reproducible state to process the input
	entropy	either frequency domain (e.g. spectral entropy) or time (e.g. sample entropy) domain	a family of measures used to quantify uncertainty, irregularity, or unpredictability in neural signals. In its classical Shannon formulation, entropy quantifies uncertainty in a probability distribution	in neural activity, higher entropy generally indicates more variable or less predictable signal structure, whereas lower entropy indicates greater regularity or constraint
	Lempel-Ziv complexity (LZC)	time	computed by binarizing or symbolizing a neural time series and estimating the number of distinct temporal patterns required to reconstruct the sequence. It indices the diversity and compressibility of the signal's temporal structure	higher LZC indicates a less repetitive, less compressible signal with a more diverse temporal pattern structure

## 3. Temporal mechanism I: temporal matching

### (a) Definition

The first mechanism is temporal matching, which refers to the initial temporal correspondence between an extrinsic stimulus and the intrinsic spontaneous activity of the brain. As an early processing step, the temporal sequences characterizing the extrinsic stimulus may be tracked and coordinated by the temporal sequences and structure of spontaneous neural activity. Consider a pair of dancing partners: during a dance, each partner tracks and coordinates their movements with both the music and the other partner's movements, matching them in a precise temporal sequence. In a similar fashion, recent empirical work suggests that the intrinsic neural rhythms and timescales of the brain can track the extrinsic rhythms and timescales of environmental stimuli [111,112]. Increasing evidence suggests that this may occur because the temporal structure of extrinsic stimuli is processed in terms of its statistical distribution or natural stochastic structure and matched by the intrinsic neural stochastic structure of the brain [111,113–115]. The concept of a stochastic structure refers to probability distributions with randomness and uncertainty, as distinguished from exact rules with complete certainty. Hence, temporal matching involves direct comparisons between the probability distributions of extrinsic inputs/stimuli, their natural stochastic structure, and the intrinsic spontaneous activity of the brain, its neural stochastic structure.

This mechanistic definition of temporal matching must be distinguished from the narrower concepts of entrainment and alignment. Entrainment refers to the brain's temporal tracking of rhythmic-periodic stimuli over short durations [90,116,117], whereas alignment describes the brain's temporal tracking of non-rhythmic aperiodic stimuli over longer durations [90,112]. Both concepts describe the observable *outcome* of a process that tracks and compares two key temporal structures: that of extrinsic stimuli and that of intrinsic spontaneous activity. Therefore, we propose temporal matching as the mechanism underlying both rhythmic-periodic entrainment and non-rhythmic aperiodic alignment to stimuli.

How then is temporal matching implemented during stimulus processing? We expect that the dynamic features of neural activity may play a key role in this mechanism. Environmental stimuli contain both rhythmic-periodic and non-rhythmic aperiodic temporal structures across different timescales. Accordingly, the brain may use its own intrinsic scale-specific oscillatory dynamics to match extrinsic rhythmic-periodic stimulus features. The brain may rely on its scale-free aperiodic dynamics to match non-rhythmic stimulus features. In this way, temporal matching may link the intrinsic neural stochastic structure of the brain with the natural stochastic structure of extrinsic environmental stimuli (figure 3).

**Figure 3.**
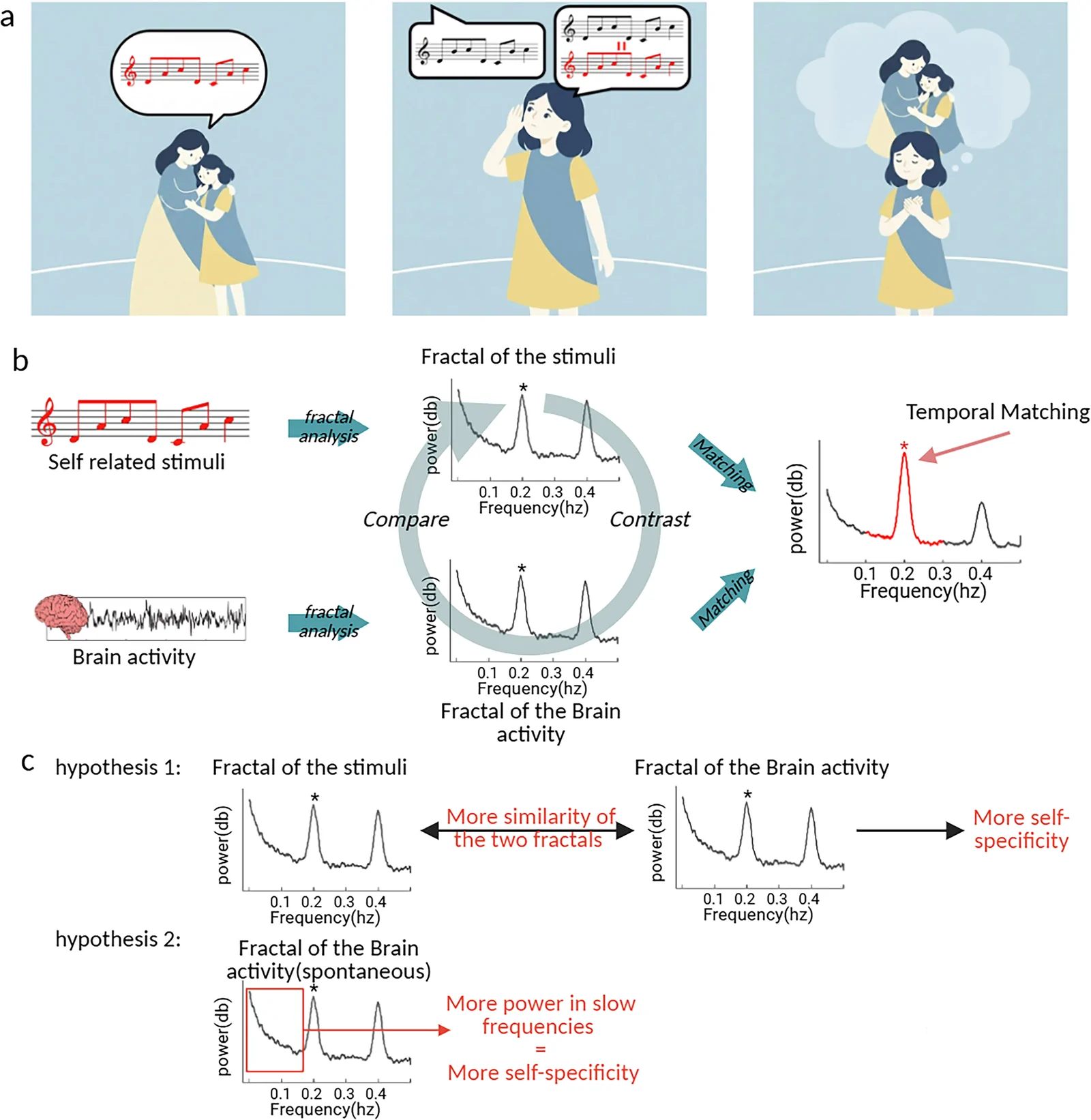
Temporal matching of extrinsic stimuli and their natural stochastic structure. (a) This panel depicts the psychological process of linking external stimuli to the self. A person's internal ‘self-representation’—formed through personal experiences and bonds (left)—acts as a template that, on the neuronal level, manifests in the neural stochastic structure of its intrinsic spontaneous activity. When extrinsic environmental stimuli are encountered with their natural stochastics, such as music (middle), the natural stochastic structure of the brain filters for the music dynamics and its stimuli that resonate with the internal template of the brain, which is manifested in its own spontaneous activity's neural stochastic structure: the better both natural stochastics and neural stochastics match and thus converge with each other, the stronger a feeling and/or memory related to the subject's self will be induced. (b) Mechanism: scale-free dynamics and stochastic matching. This panel breaks down the underlying neuronal mechanism: fractal analysis: both self-related stimuli (like music) and spontaneous brain activity exhibit scale-free dynamics. Through its own degree of scale-free dynamics (measured by the power law exponent (PLE); see table 2), the brain ‘compares’ and thus matches its own intrinsic neural stochastic structure (internal noise and rhythms) with the natural stochastic structure of the extrinsic environment. When the scale-free dynamic fractal peaks of the natural stochastics of external stimuli align with the intrinsic patterns of the brain and its neural stochastics, ‘temporal matching’ occurs. (c) Hypothesis 1: greater similarity between the fractal (or stochastic) structures of extrinsic stimuli and spontaneous brain activity leads to stronger temporal convergence, thereby predicting higher degrees of self-association to extrinsic stimuli. Hypothesis 2: stronger intrinsic scale-free dynamics—characterized by dominant power in slow frequencies (higher PLE)—actively modulate the matching process, exerting a stronger ‘pull’ on extrinsic stimuli to increase their association with higher degrees of self-specificity.

### (b) Hypotheses

#### (i) Hypothesis I: temporal matching predicts stimulus self-association

We hypothesize that the degree of temporal matching between the intrinsic neural stochastic structure of the brain and the natural stochastic structure of extrinsic stimuli predicts the degree of self-association, or self-specificity, of those stimuli. More specifically, the stronger the convergence between the scale-specific and/or scale-free dynamics of an extrinsic stimulus and the corresponding neural dynamics (scale-specific and/or scale-free) of spontaneous brain activity, the more strongly intrinsic self-representation may be recruited. This, in turn, should increase the likelihood that the stimulus is associated with a higher degree of self-specificity. Accordingly, extrinsic stimuli whose temporal structure more closely resembles the temporal structure of a participant's intrinsic spontaneous brain activity should show stronger self-association.

#### (ii) Hypothesis II: resting-state scale-free dynamics modulate temporal matching

Whereas hypothesis I focuses on how the similarity between the natural stochastic structure of extrinsic stimuli and the neural stochastic structure of intrinsic spontaneous activity predicts stimulus self-association, hypothesis II concerns the *active influence* of the intrinsic spontaneous activity of the brain on temporal matching itself.

More specifically, we hypothesize that stronger scale-free dynamics in intrinsic spontaneous activity, reflected in a higher PLE (table 2), are associated with a greater likelihood of self-association to extrinsic stimuli. A higher PLE indicates relatively stronger power at slower frequencies than at faster frequencies. This stronger slow-frequency dominance may allow spontaneous activity to exert greater influence on the temporal matching between intrinsic neural dynamics and the natural stochastic structure of extrinsic stimuli. Figuratively, extrinsic stimuli may be more strongly ‘pulled’ toward the self and its intrinsic self-representation, resulting in stronger self-association. Accordingly, individual differences in the resting-state PLE may lead different participants to temporally match the same extrinsic stimuli in different ways, resulting in distinct degrees of self-association across individuals.

#### (iii) Empirical evidence for hypothesis 1

Hypothesis I, concerning the temporal matching between extrinsic stimuli and intrinsic brain activity, is supported by several empirical findings. Evidence from fMRI [90,114] and EEG [118] indicates that the intrinsic scale-free dynamics of the brain follow the scale-free dynamics and timescales of naturalistic environmental stimuli. For instance, Klar *et al.* [90] demonstrated that the temporal structure of extrinsic stimuli (2 s duration), presented in intertrial intervals of 52–60 s, leads to peaks in the brain's power spectrum at exactly that same frequency range (0.016 to 0.019 Hz) while importantly, maintaining its scale-free dynamics. Such dynamic and stochastic relationships between the frequency of extrinsic stimuli and the intrinsic frequency of the brain (measured by scale-free dynamics) have been observed in several studies across both slower (fMRI) ([91,114,116,117,119]) and faster (EEG) ([120,121]) frequency ranges.

Moreover, the empirical findings support the key role of spontaneous activity in self assumed in both hypotheses I and II. Spontaneous activity and especially its scale-free dynamics have been linked to several facets of the self, including self-consciousness [7,81], self-identity [84], task-related self-continuity [6], and self-prioritization [5]. These findings suggest the existence of an intrinsic self-representation that is predominantly grounded in spontaneous activity and its intrinsic scale-free dynamics [1,48]. Accordingly, intrinsic self-representation may be mediated primarily by the relationships among different frequency bands and their timescales, as captured by scale-free dynamics and quantified by the PLE, rather than by scale-specific dynamics within single frequency bands such as alpha, theta, or gamma [7]. Further indirect support for this view comes from the lack of evidence for one specific frequency band underlying self-association with extrinsic stimuli: alpha [49], delta [103], theta [59,103], gamma [54,57,60,122,123], and beta [58,59] bands have all been implicated in self-association and self-related cognition [29,61].

#### (iv) Empirical evidence for hypothesis 2

Support for hypothesis 2 comes from findings suggesting that spontaneous and pre-stimulus activity can actively shape self-association with extrinsic stimuli, although direct evidence for the specific role of scale-free dynamics remains limited. This active influence can be observed in both pre-stimulus and resting-state activity. Pre-stimulus spontaneous activity has been shown to mediate self-association with extrinsic stimuli through several neural features: pre-stimulus amplitude levels in the ventromedial prefrontal cortex and other CMS in fMRI [1,124,125], pre-stimulus alpha-band amplitude [48], and pre-stimulus fronto-occipital functional connectivity in EEG [48,90]. Analogous findings have been reported for resting-state activity. Higher resting-state PLE and longer intrinsic neural timescales (INT), measured by ACW (table 2), have been associated with stronger self-association with extrinsic stimuli [5,6,8]. This view is further supported by substantial inter-individual differences in these measures, including resting-state PLE [5,7,81] and early event-related potentials (ERPs), such as N1, which may reflect individual differences in self-association with extrinsic stimuli or self-prioritization [5,48,64]. Together, these findings support the broader claim that spontaneous activity, through its intrinsic self-representation, shapes self-association with extrinsic stimuli.

### (c) Open questions and testable predictions

Both spontaneous PLE and ERP measures, such as N100 and N170/200, have been associated with the degree of self-association to extrinsic stimuli [37,38,40–44,63,126–128]. However, whether and how the intrinsic spontaneous activity's scale-free dynamics are related to ERP markers during self-association with extrinsic stimuli remain open questions. This leads to the testable prediction that higher spontaneous activity PLE, reflecting a stronger intrinsic basis for temporal matching between intrinsic self-representation and extrinsic stimuli, may be associated with stronger ERP modulation during self-association to extrinsic stimuli, including higher N100 amplitude [40–44,126,128] and lower N170/N200 amplitude [37,38,63,127,128].

Furthermore, temporal matching with different types of stimuli may relate to distinct concepts of the self. Stimuli can be broadly classified into three categories: interoceptive stimuli, which originate within the body; exteroceptive stimuli, which originate in the external environment; and cognitive stimuli, which may originate within neural activity itself. Despite their different origins, temporal matching may operate similarly across all three stimulus types (in a domain-independent or domain-general way; [129]) by matching their respective stochastic structures with the neural stochastic structure of spontaneous activity [130]. Thus, temporal matching may provide a common mechanism for quantifying self-association across distinct dimensions or domains of the self: interoceptive stimuli may relate primarily to the bodily or minimal self [56,131], exteroceptive stimuli to the ecological or social self [22,29,30,33], and cognitive stimuli to the cognitive, narrative, or autobiographical self [13,25,63].

Finally, one may ask whether and how temporal matching between stimuli and brain activity relates to meaningful stimulus information. Kumar *et al.* [115] provide a first step in this direction by examining semantic fluctuations in speech at the word level using WordNet and sentences using SBERT. They calculated semantic timescales using a dynamic ACW and applied the same dynamic ACW approach to neural activity. Their findings revealed that fluctuations in semantic timescales, measured by semantic ACW, were causally related to fluctuations in neural timescales, particularly in language regions, as assessed using transfer entropy. These findings provide initial evidence that temporal matching may link semantic and neural timescales and support the brain's dynamic tracking of semantically meaningful information. As a next step, investigating whether the PLE of intrinsic spontaneous activity modulates the relationship between extrinsic stimuli and intrinsic neural timescales could help ground temporal matching in the scale-free dynamics of the brain and its intrinsic self-representation.

## 4. Temporal mechanism II: temporal integration

### (a) Definition

Following temporal matching between extrinsic stimuli and the brain's intrinsic spontaneous activity, the matched stimuli must be further processed in relation to intrinsic self-representation. We propose that this occurs through a second temporal mechanism: temporal integration. Temporal integration refers to the process by which the brain combines discrete, transient stimulus-related activity patterns across time into a coherent and temporally extended perceptual or cognitive event. Going beyond the initial stochastic matching between extrinsic stimuli and intrinsic brain dynamics, temporal integration enables post-stimulus information accumulation within a shared temporal window [98]: this may allow self-related information features of intrinsic self-representation to be integrated with the information of extrinsic stimuli such that the stimuli are perceived as self-specific [83,97]. We therefore assume temporal integration to be a key mechanism through which intrinsic self-representation and extrinsic stimuli are connected within a common temporal window.

The INTs of the brain may constitute a key dynamic feature for temporal integration (table 2). Timescales describe the duration over which a process unfolds; for example, a heartbeat has a relatively short duration of approximately 1 s, while breathing unfolds over a longer window of approximately 8–20 s. Similarly, spontaneous brain activity can be characterized by INTs of different lengths [83,97]. These timescales can be measured during task states using TRWs and during both the resting state and the task state from ACWs [83,97]. Thus, INTs provide temporal windows within which information from different time points can be combined: longer windows allow information to be integrated across longer temporal distances, whereas shorter windows promote temporal segregation across shorter intervals [83,97]. Accordingly, longer INTs may allow extrinsic stimulus-related activity to be integrated with a broader span of ongoing intrinsic spontaneous activity, thereby increasing the opportunity for intrinsic self-representation to shape the subsequent self-association of the extrinsic stimulus. Shorter INTs, by contrast, may restrict integration to narrower temporal windows, promoting temporal segregation rather than integration. This mechanism is summarized in figure 4.

**Figure 4.**
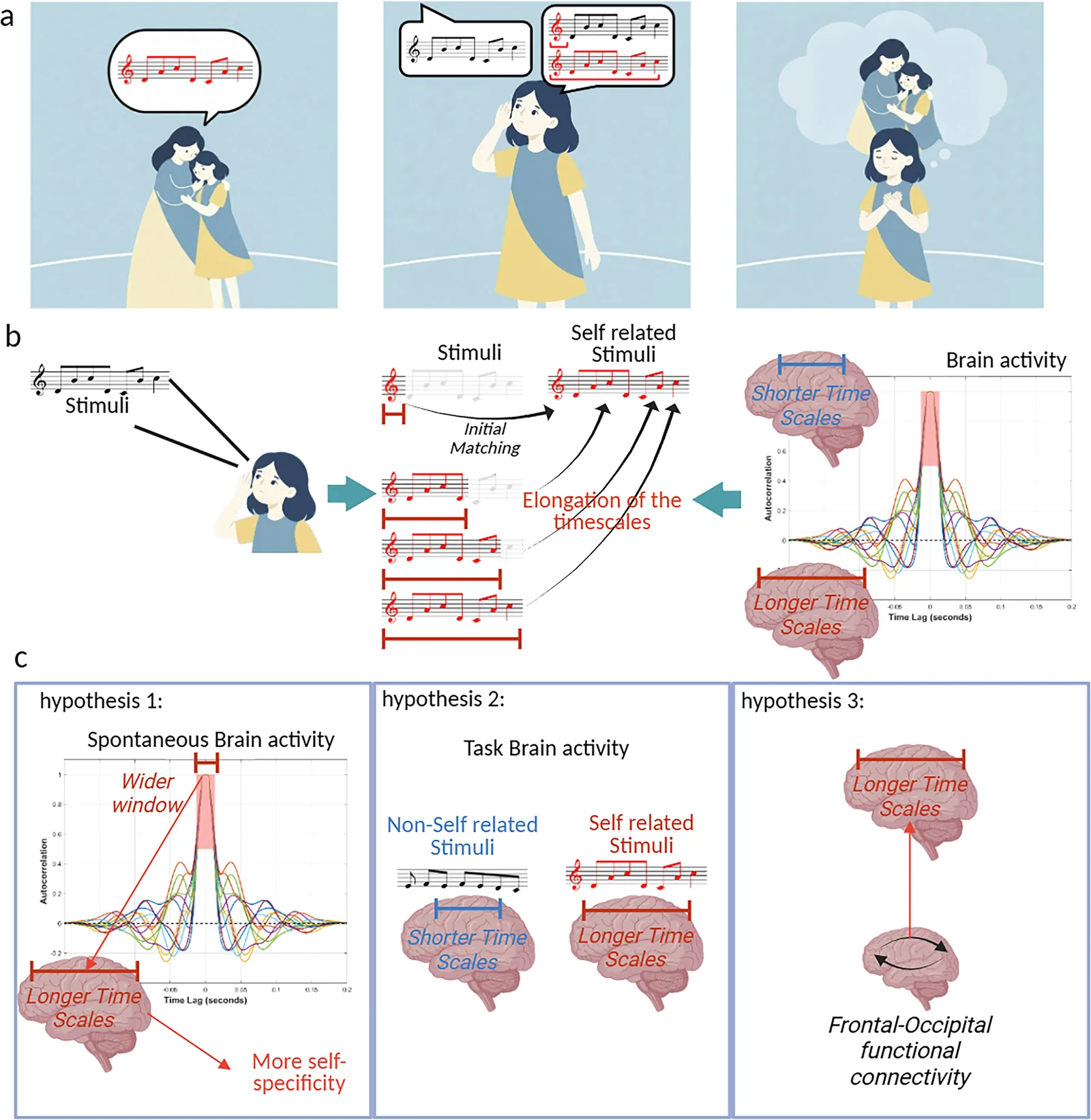
Temporal integration of the shorter duration of extrinsic stimuli within the longer duration of the intrinsic neural timescale of the brain. (a) An external stimulus, such as a melody, is not just processed in isolation; it is integrated within the brain's ongoing intrinsic self-representation (left), allowing the listener to associate the music with personal meaning or memories (right). (b) Mechanism: this panel details how the brain's intrinsic neural timescales (INT) facilitate such temporal integration: the brain possesses a variety of ‘durations’ for processing information, ranging from short to long. The autocorrelation window (ACW) measures how long a piece of neural information remains correlated with itself over time. When a stimulus is perceived as self-related, the brain's timescale (ACW) elongates or stretches; this transient widening of the brain's temporal window allows the extrinsic stimulus to be transiently ‘captured’ and temporally integrated within the continuous, long-lasting stream of the intrinsic self-representation. (c) Hypothesis 1: a wider resting-state ACW yields longer neural timescales. This expanded temporal window allows for greater accumulation of intrinsic self-specific information, directly predicting higher self-specificity. Hypothesis 2: task-state ACW significantly elongates during the processing of self-related stimuli compared to non-self-related stimuli. Hypothesis 3: enhanced frontal‒occipital functional connectivity—mediated by alpha-band dynamics—acts as a key modulatory factor that supports and sustains these longer temporal windows during self-association.

### (b) Hypotheses

#### (i) Hypothesis I: temporal integration during the resting state

First, we hypothesize that the length of the intrinsic spontaneous activity INTs is directly related to the strength of the intrinsic self-representation. Longer INTs may provide broader temporal windows for the temporal integration and accumulation of information within spontaneous activity, which we postulate contains self-specific information. Accordingly, longer resting-state ACWs should be associated with greater integration of self-specific information across time, particularly self-related features encoded over longer durations. This relationship can then be characterized by self-related psychological measures such as self-consciousness and self-identity.

#### (ii) Hypothesis II: temporal integration during task-related activity

Second, we hypothesize that task-state ACW will be longer for self-specific stimuli than for non-self-specific stimuli. Longer temporal windows may allow extrinsic stimulus-related activity to be more strongly integrated with intrinsic self-representation, thereby increasing the degree to which the extrinsic stimulus is associated with self-specificity. In this context, we expect the duration of ACW to be related to standard electrophysiological indices of task-related self-processing, including the N100, N170/N200, and P300. Specifically, longer ACW in both the resting and task states, as well as stronger rest-task ACW prolongation, should be associated with higher N100 amplitudes [40–44,126,128], lower N170/N200 amplitudes [37,38,63,127,128], and higher P300 amplitudes and/or later event-related potentials [98,132,133] for stimuli with high self-specificity than for those with low self-specificity. We propose that longer ACW windows reflect the degree to which intrinsic self-representation is integrated with extrinsic stimulus processing such that stimuli integrated over longer temporal windows are more likely to acquire self-specificity.

#### (iii) Hypothesis III: the role of alpha band dynamics in temporal integration

Third, given the role of alpha activity in mediating pre- and post-stimulus activity more generally [134] and self-specific processing more specifically ([72,79]), we hypothesize that ACW is related to alpha-band dynamics during self-association with extrinsic stimuli. Longer ACW during resting and task states may be associated with stronger prefrontal‒occipital alpha functional connectivity during both pre- and post-stimulus periods [72], as well as higher pre-stimulus alpha power [79]. These alpha-band dynamics may facilitate stronger self-association with extrinsic stimuli. Accordingly, we hypothesize a three-way relationship among the resting-state ACW, rest-task ACW prolongation, and stimulus self-association, with alpha-band activity across the pre- and post-stimulus periods serving as a key modulatory factor ([134,135]).

### (c) Empirical evidence

Research on language processing has demonstrated that the brain's TRWs vary according to the duration of extrinsic stimuli [50,98,103,136]. Single words are associated with shorter neural TRWs, whereas longer and more complex stimuli, such as sentences or paragraphs, are associated with longer TRWs in higher-order regions, including the default-mode network [50,132,133]. Moreover, the dynamic fluctuations in the semantics of speech, measured by its timescale (semantic ACW), directly and causally (measured by transfer entropy) relate to the fluctuations in the brain's INT (measured by neural ACW) [115]. These findings suggest that neural timescales are differentially recruited to integrate stimuli across varying temporal durations during task-related activity.

Building on this, studies have shown that the duration of neural timescales during task states is related to the degree of self-specificity of the extrinsic stimulus. ACW has been found to change from rest to task states, with longer task-state ACW relative to rest associated with higher degrees of self-association to extrinsic stimuli [8,83]. These findings suggest that longer ACW during task-related processing may support the temporal integration of transient extrinsic stimuli with ongoing intrinsic self-representation. Specifically, longer task-related ACW may provide broader temporal windows in which extrinsic stimulus-related activity can be integrated with ongoing spontaneous activity, increasing the likelihood that the stimulus is assigned self-specific significance [8,83]. By extension, resting-state studies have shown that longer spontaneous ACW is associated with higher levels of self-consciousness and self-association [5,7,8,137].

Together, these results provide a foundation for hypotheses I and II: INT correspond to the temporal duration of extrinsic stimuli and are associated with the degree of self–stimulus association. The broader relationship between longer INTs and self-related psychological measures further suggests that extended temporal integration may support not only the processing of stimulus complexity but also intrinsic self-representation. Hypothesis III remains to be tested.

### (d) Open questions and testable predictions

An important open question is whether longer INTs in the DMN provide a temporal basis for intrinsic self-representation. INTs follow a topographical gradient from unimodal to transmodal regions, with shorter timescales in unimodal sensorimotor, exteroceptive, and interoceptive regions and longer timescales in transmodal regions, including the DMN [83,97,138,139]. Because the DMN overlaps with regions implicated in the narrative self [140], mental self [25] and autobiographical self [13], its longer temporal windows may support the accumulation and integration of information required for higher-order self-related processes. This finding is consistent with the role of the DMN in autobiographical memory, mental simulation, prospection, retrospection, theory of mind and mind-wandering [141–144]. Future studies should test whether longer DMN INTs, measured by ACW or TRW, are associated with stronger intrinsic self-representation and with psychological measures indexing narrative, autobiographical, or mental selfhood.

Extending this topographical gradient from uni- to transmodal regions, regional differences in temporal integration may help distinguish different layers of self [25]. Shorter temporal windows in lower-order unimodal regions may support the processing of shorter-duration interoceptive and exteroceptive stimuli associated with the bodily or minimal self. Longer temporal windows in higher-order transmodal regions, especially the DMN, may support the integration of more temporally extended cognitive information associated with the mental, autobiographical, and narrative self. Although tentative, this suggests that different layers of self (interoceptive-bodily, exteroceptive, cognitive or mental) may be partly grounded in regionally specific timescales of temporal integration (see the proposed framework below and boxes 1 and 2 for details on different concepts of the self).

## 5. Temporal mechanism III: temporal differentiation

### (a) Definition

In the preceding sections, we described temporal matching as the coordination of extrinsic stimulus natural stochastics with the intrinsic spontaneous activity's neural stochastics of intrinsic self-representation, followed by temporal integration, in which shorter stimulus-specific durations are integrated within the longer INTs associated with intrinsic self-representation. However, despite this integration, extrinsic stimuli, including their degree of self-specificity, continue to be perceived as distinct from intrinsic self-representation. We explain this distinction through our third mechanism: temporal differentiation (figure 5).

**Figure 5.**
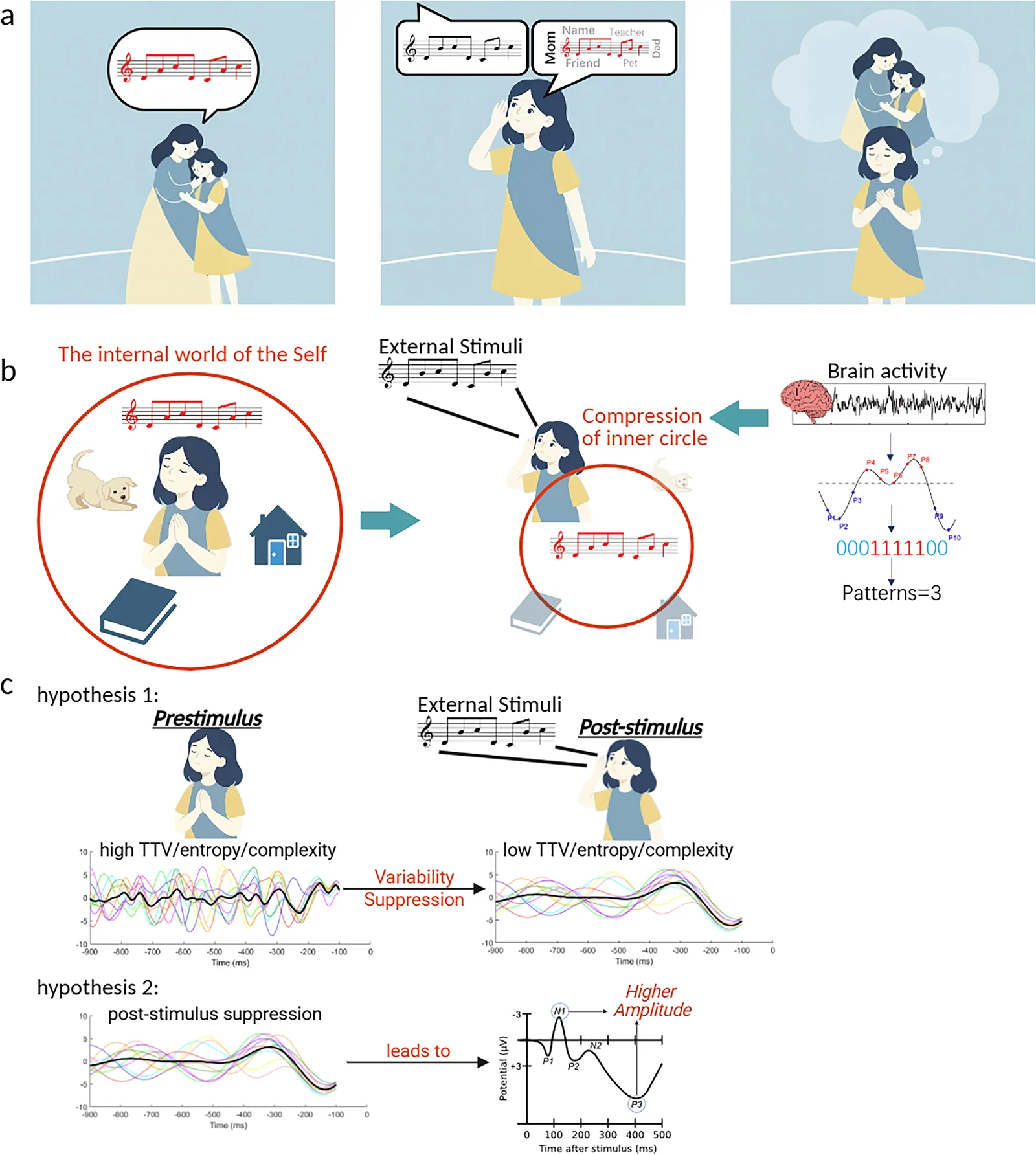
Transient temporal differentiation of the short-duration extrinsic stimuli's self-association from the long-duration intrinsic self-representation. (a) The extrinsic stimulus triggers internal associations (memories, feelings of ‘mother’), allowing the extrinsic stimulus or input to be matched with the intrinsic self-representation and retained as a self-specific experience. (b) Neural mechanism: a schematic of the interaction between intrinsic spontaneous activity and extrinsic activity task-related activity. The ‘internal world of the self’ represents the ongoing, stable and wide-ranging intrinsic self-representation as manifest in spontaneous activity, including pre-stimulus activity. When ‘external stimuli’ are introduced, the intrinsic spontaneous activity undergoes transient suppression and compression (the red circle around the notes). This transient compression distinguishes the short-term extrinsic stimulus from the long-term intrinsic self-representation. The right panel illustrates the measurement of this process using trial-to-trial variability (TTV), Lempel–Ziv complexity (LZC) and entropy; lower post-stimulus complexity reflects the successful compression of the signal required to perceive the stimulus as self specific. (c) Hypothesis 1: highly complex, variable, and entropic pre-stimulus activity undergoes a sharp transient post-stimulus reduction. This transient suppression and compression allow the short-term self-associated stimulus in the foreground to be transiently temporally differentiated from the continuous background of the intrinsic self-representation. Hypothesis 2: greater post-stimulus suppression directly leads to enhanced evoked responses (higher N100 and P300 amplitudes). This relationship demonstrates that, as the intrinsic background with its intrinsic self-representation recedes transiently during the post-stimulus period, the self-specificity of the extrinsic stimulus shifts more strongly into the foreground of our perception.

Temporal differentiation refers to the process by which different neural processes are separated on the basis of their temporal features, such as their change over time. In the present context, temporal differentiation concerns the neural and psychological distinction between transient, extrinsic stimulus-evoked self-association during the post-stimulus period, on the one hand, and the more continuous intrinsic self-representation related to spontaneous activity, on the other hand. The situation becomes even more complex given that spontaneous activity is carried over to the post-stimulus period through the pre-stimulus activity. This pre-to-post-stimulus carry-over allows intrinsic self-representation to interact directly with extrinsic stimuli at stimulus onset. We propose that this interaction enables the extrinsic stimulus and its degree of self-association to be differentiated from intrinsic self-representation on temporal grounds. Operationally, this differentiation may be reflected in short-term post-stimulus reductions in neural variability, measured by TTV, as well as reductions in irregularity or complexity, measured by entropy and LZC (see table 2).

### (b) Hypotheses

#### (i) Hypothesis I: temporal differentiation operates through transient suppression of ongoing spontaneous activity dynamics during self-associated stimulus processing

First, we hypothesize that intrinsic self-representation within spontaneous activity shapes the processing of extrinsic stimuli through its influence on the pre-stimulus period. During the post-stimulus period, this ongoing spontaneous activity is transiently suppressed and compressed, allowing shorter-lasting extrinsic stimuli and their self-association to be temporally differentiated from the more continuous intrinsic self-representation. Neuronally, this transient suppression should be reflected in reduced post-stimulus variability, irregularity and complexity relative to the pre-stimulus period. Specifically, stronger self-association should be associated with greater TTV suppression, reflected in lower post-stimulus TTV; greater entropy reduction, reflected in lower post-stimulus entropy; and greater complexity compression, reflected in lower post-stimulus LZC. These transient post-stimulus reductions may allow the self-associated stimulus to emerge more distinctly in perception and memory against the background of ongoing intrinsic self-representation.

#### (ii) Hypothesis II: temporal differentiation is associated with perceived self-specificity and event-related potential responses

Second, we hypothesize that post-stimulus reductions in amplitude variability, irregularity and complexity, reflected in lower TTV, entropy and LZC, index the transient suppression of the longer-lasting and more stable intrinsic self-representation during a short post-stimulus window of approximately 500–1000 ms. At the electrophysiological level, we predict a negative relationship between ERP amplitude and post-stimulus levels of TTV, entropy and LZC. Higher N100 and P300 amplitudes, reflecting stronger foreground perception of the extrinsic stimulus’ self-specificity, should be associated with lower post-stimulus TTV, entropy and LZC, reflecting greater transient suppression of intrinsic self-representation in the perceptual background [82,117]. Conversely, when expressed as reduction relative to the pre-stimulus period, stronger post-stimulus reductions in TTV, entropy and LZC should be associated with higher ERP amplitudes and stronger perceived self-specificity. This transient suppression allows the self-specificity of the extrinsic stimulus to become foregrounded in perception, while the intrinsic self-representation transiently recedes into the background compared with both the resting state and pre-stimulus period. Consequently, higher N100 and P300 amplitudes, coupled with greater post-stimulus reductions in TTV, entropy and LZC, will jointly index stronger perceived self-specificity of the extrinsic stimulus.

#### (iii) Empirical evidence for hypotheses I and II

Various studies have shown that intrinsic neural activity in the 500 ms window preceding stimulus onset significantly influences how the extrinsic stimulus is subsequently processed during the 500–1000 ms post-stimulus period [82,104,134]. Both higher pre-stimulus amplitude and variability in the alpha band have been shown to be related to lower trial-to-trial variability (TTV; table 2) and increased ERP amplitudes (N100, P300) during the post-stimulus period [82,104,134]. These findings suggest that pre-stimulus signal properties shape post-stimulus neural differentiation.

Moreover, phase-related processes may also be involved: extrinsic stimuli show higher inter-trial phase coherence (ITPC) at stimulus onset in trials with higher pre-stimulus variability and entropy [82,121,127,145]. In other words, higher pre-stimulus irregularity may allow for more temporally precise stimulus processing at stimulus onset and during the post-stimulus period (higher ITPC); we assume that this is owing to the fact that higher pre-stimulus complexity, variability and entropy can be more strongly transiently suppressed by the extrinsic stimulus at stimulus onset when compared to their low pre-stimulus values. Similarly, using LZC, higher pre-stimulus complexity (−500 to 0 ms) was found to be associated with lower post-stimulus complexity and higher ERP amplitude during the post-stimulus period (0 to 500/1000 ms), compared with lower pre-stimulus complexity [82,121]. Similarly, Avramescu *et al.* [146] reported that, during a self-recognition task, smaller rest-task increases in neural entropy were associated with lower entropy in response times for ambiguous stimuli. Overall, these three lines of evidence demonstrate that when task activity is characterized by strong post-stimulus reduction of the intrinsic pre-stimulus spontaneous dynamics (TTV, LZC, or entropy), subsequent task performance and stimulus-related information processing are more highlighted and focused, resulting in stronger post-stimulus activity (higher ERP, higher ITPC, lower TTV, LZC and entropy) and more efficient behaviour/perception (faster reaction times, higher accuracy).

#### (iv) Empirical evidence for non-additive pre-to-post-stimulus interaction

Together, these results illustrate a non-additive interaction of pre- and post-stimulus activity [82,104,134]. Under a purely additive account, the effects of an extrinsic stimulus would simply be superimposed on the level of pre-stimulus activity, such that higher pre-stimulus amplitude variability, entropy, or complexity should lead to higher post-stimulus levels of the same measures. Empirically, however, the opposite pattern is often observed: higher pre-stimulus levels are followed by lower post-stimulus levels [134,147]. This suggests that extrinsic stimuli do not merely add to ongoing spontaneous activity, but interact with it in a way that transiently suppresses or compresses aspects of the post-stimulus signal [82,89,105,106,148,149]. The precise form of this interaction, however, probably depends on task demands, stimulus ambiguity, and the measure considered.

In the context of self-association, such non-additive pre-to-post-stimulus interactions may support temporal differentiation by allowing extrinsic self-specific stimuli to emerge against the background of intrinsic self-representation. We therefore hypothesize that the degree of non-additive pre-to-post-stimulus interaction is stronger for stimuli with higher self-specificity than for stimuli with lower self-specificity. Several lines of evidence are consistent with this hypothesis. First, perceived stimulus self-specificity has been associated with higher post-stimulus N100 and P300 amplitudes (see table 1 for various lines of evidence). Second, pre-stimulus activity, including its variability, entropy, and complexity, has been shown to modulate subsequent self-specific stimulus processing [77,79,150]. Third, self-specific task conditions have been associated with lower entropy and lower LZC [82,121], while lower LZC has also been observed in CMS/DMN regions implicated in self-association [151]. Overall, self-specific stimuli may be accompanied by stronger transient suppression or compression of ongoing activity, supporting their temporal differentiation from intrinsic self-representation.

Given that entropy indices signal irregularity and LZC indices signal complexity or compressibility, we propose that their transient reduction during the post-stimulus period reflects a transient suppression or compression of intrinsic self-representation during its interaction with the extrinsic stimulus at stimulus onset. This neural account may also have a psychological counterpart. In psychology, temporal compression refers to the tendency for objects or events to be represented with less specificity and greater abstraction as their temporal distance from the present increases, making them less distinguishable from other events over time [152]. Accordingly, as the extrinsic stimulus becomes more temporally distant from its moment of occurrence, its content may become increasingly compressed and abstracted. We hypothesize that this abstraction may facilitate the attribution of higher degrees of self-specificity to the extrinsic stimulus [73,153].

### (c) Open questions and testable predictions

First, there is currently no direct evidence that the degree of self-association of extrinsic stimuli is related to the non-additivity of the pre-to-post-stimulus interaction. On the basis of the literature discussed above, we tentatively propose a relationship between these two properties such that stronger self–stimulus association is related to stronger non-additive pre-to-post-stimulus dynamics. Specifically, pre-to-post-stimulus reductions in TTV, entropy, and LZC may reflect a transient compression of ongoing spontaneous activity, allowing self-associated stimuli to become more perceptually salient against the background of intrinsic self-representation. In this sense, temporal differentiation does not imply that self-specific stimuli are less related to intrinsic self-representation; rather, it refers to the process by which extrinsic self-associated stimuli are rendered distinct in the foreground of our perception as embedded within an ongoing intrinsic self-representation in the background.

Second, the relationship between neural compression or differentiation and psychological temporal compression remains largely unexplored. We hypothesize that transient post-stimulus reductions in TTV, entropy and LZC may be related to psychological abstraction, such that greater neural compression during self-related stimulus processing would be associated with greater temporal compression of the perceived stimulus. In this view, self-related stimuli may become more perceptually salient while being represented in a more compressed or abstracted form over time. However, direct evidence for this neural‒psychological relationship is currently lacking, particularly for LZC. Related evidence comes from Avramescu *et al.* [146], who reported that reduced rest-task increases in neural entropy during a self-recognition task were associated with reduced response time (RT) entropy for ambiguous self-other morphs. One possible interpretation is that lower RT entropy reflects more focused behavioural information processing when ambiguous extrinsic stimuli are evaluated against a more stable intrinsic self-representation. However, whether this behavioural focusing corresponds to psychological temporal compression or abstraction remains an open question. Future studies should directly test whether transient post-stimulus reductions in entropy and LZC are associated with psychological temporal compression, abstraction, and perceived self-specificity.

## 6. Our proposed framework: a temporal, dynamic model of self and the brain

### (a) Hierarchical and temporally nested organization of the three mechanisms

How are the three temporal mechanisms related to each other? We propose that temporal matching, temporal integration, and temporal differentiation form a hierarchical and temporally nested sequence of processing stages through which extrinsic stimuli become associated with intrinsic self-representation.

At the bottom of the hierarchy, temporal matching constitutes the most basic mechanism. It allows the temporal structure of extrinsic stimuli to be matched with the brain's intrinsic spontaneous activity through scale-free dynamics, indexed by PLE, and scale-specific oscillatory dynamics. Without such matching between the natural stochastic structure of extrinsic stimuli and the intrinsic neural stochastic structure of spontaneous activity, extrinsic stimuli cannot be related to intrinsic self-representation. In this sense, temporal matching may be considered the first layer of self-associative processing and a necessary neural condition for self-association with extrinsic stimuli.

However, temporal matching alone is insufficient. For extrinsic stimuli to acquire self-specificity, they must also be integrated with intrinsic self-representation. This is achieved in the next hierarchical layer through temporal integration, mediated by INT such as the ACW. Through these temporal windows, the shorter durations of extrinsic stimuli can be integrated within the longer temporal windows of intrinsic self-representation. Temporal integration therefore builds on temporal matching: whereas temporal matching allows extrinsic stimuli and spontaneous activity to be compared through similarities in their respective stochastic structures, temporal integration directly connects them within a shared temporal window. Temporal integration may therefore be considered a sufficient neural condition for self-association with extrinsic stimuli.

However, temporal integration leaves open how self-associated stimuli are perceived as distinct in the short-lasting foreground of our perception as embedded within its longer-lasting background of intrinsic self-representation. This is addressed by temporal differentiation. At the top of the hierarchy, temporal differentiation allows a temporally integrated extrinsic stimulus to be distinguished from pre-stimulus activity and its intrinsic self-representation through transient reductions in amplitude variability, irregularity, and complexity, indexed by TTV, entropy and LZC. In this way, self-associated stimuli may become perceptually salient in the foreground, while intrinsic self-representation is transiently suppressed and thus delegated to the background in our perception. Temporal differentiation may therefore be considered a sufficient neural condition for perceiving self-associated stimuli as distinct perceptual objects within an ongoing self-related background.

This functional hierarchy may also be specified in terms of processing duration. Temporal matching operates across the broadest range of timescales, from shorter rhythmic or entrainment-based timescales to longer aperiodic timescales involved in tracking complex environmental sequences, such as speech or music [114,115]. This broad range is consistent with the role of scale-free dynamics. Temporal integration operates over more restricted intermediate timescales, constrained by the duration of INT such as ACW, which vary across faster EEG/MEG frequency ranges and slower fMRI frequency ranges [83,97]. Finally, temporal differentiation operates over the shortest timescales, typically within the millisecond range of pre- and post-stimulus activity [82,106,134].

Accordingly, the sequence from temporal matching to temporal integration to temporal differentiation may be characterized by a continuum from longer over intermediate to shorter processing durations. This suggests a temporally nested organization: the shorter timescale of temporal differentiation is embedded within the broader timescale of temporal integration, which is itself embedded within the broader timescale of temporal matching. Although speculative, this model suggests that the hierarchy of temporal mechanisms is intrinsically temporal: a nested hierarchy of processing durations through which extrinsic stimuli are matched with, integrated into, and perceptually distinguished against intrinsic self-representation. In this way, the nested temporal hierarchy may allow extrinsic stimuli not only to become associated and merged with the intrinsic self but also to be organized and interpreted as sources of self-related information.

### (b) From a nested hierarchy of temporal processing to dynamic models of self and the brain

This nested hierarchy of temporal self-association may have major implications for modelling both intrinsic and extrinsic manifestations of the self. If intrinsic self-representation and stimulus-evoked self-association are connected through temporal mechanisms operating across different timescales, then the self may be understood as being temporally layered rather than being determined only by its spatial localization in the brain and its cognitive content and information. Specifically, intrinsic self-representation appears to unfold across much longer timescales, spanning extended periods of one's life [82], whereas extrinsic self–stimulus association is transient and operates over much shorter timescales, from milliseconds to seconds (see table 1). However, it remains unclear how the temporal nature of the intrinsic self-representation interacts with its more content-defined conceptualizations, such as self-identity, bodily self, narrative self, social self, agency, and ownership (see boxes 1 and 2).

The temporal hierarchy of the intrinsic self-representation and its three temporal mechanisms of self-association to extrinsic stimuli are embedded within the various layers of the brain at different timescales, as recently proposed in the dynamic layer model of the brain (DLB) [65,154]. Roughly, the DLB characterizes the brain as a nested hierarchy of processing layers operating across different timescales, from longer-duration spontaneous activity in the neural background to shorter-duration task-related activity in the neural foreground [65,154]. In this framework, the brain is understood primarily by its overarching temporal–dynamic and spatial–topographical organization rather than in terms of isolated measures of cognitive contents, discrete regions, networks, frequencies or isolated timescales. This broader spatiotemporal view of the brain has been proposed to shape and thus be relevant for mental features such as the self, thought, episodic memory, and consciousness [1,65,95,145,155–157]. Accordingly, the DLB as the core of what we recently introduced as ‘spatiotemporal neuroscience’ ([65,154,158,159]) provides a broader framework for the present temporal model of self, in which self-association is understood as a nested and layered hierarchy of mechanisms operating across different processing durations (see figure 6 for the schema).

**Figure 6.**
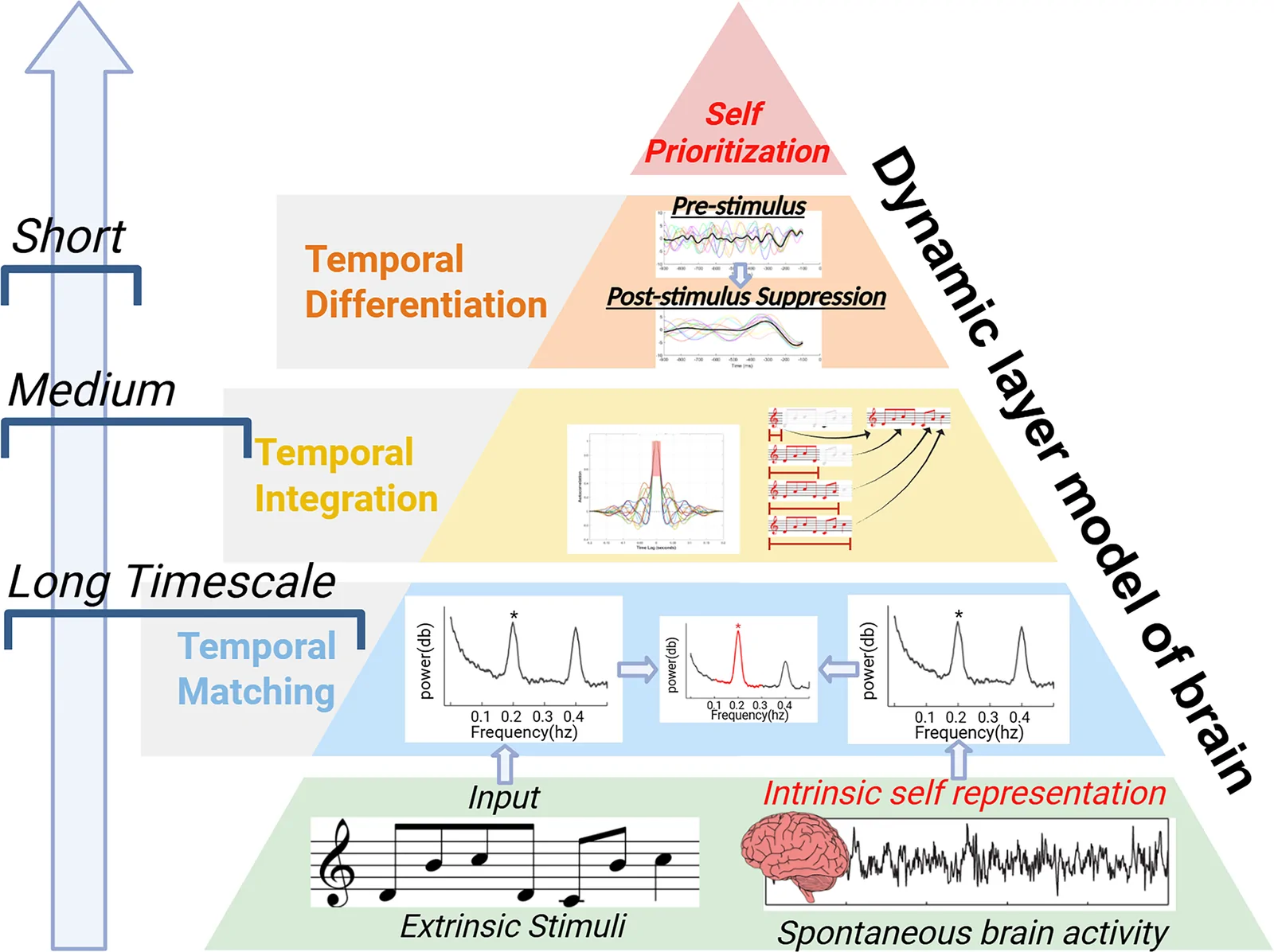
Hierarchical and temporally nested organization of the three mechanisms. The schematic illustrates the sequential temporal processing stages through which extrinsic stimuli become associated with the brain's intrinsic self-representation (bottom layer, right), resulting in self-prioritization (top layer). At the foundation, temporal matching operates across broad, long timescales to align the stochastic structures of external inputs with intrinsic spontaneous brain activity—this is mediated by scale-free dynamics as manifested in the power spectrum and its peaks at the stimulus-related frequency. Building upon this, temporal integration functions over intermediate timescales to embed shorter stimulus durations within the longer intrinsic temporal windows of the brain (e.g. measured by the autocorrelation window (ACW)). Finally, temporal differentiation operates at the shortest timescales to perceptually distinguish the temporally integrated stimulus from the background neural activity via transient variability suppression (e.g. reduced temporal variance, entropy, and compression/complexity) during the post-stimulus period relative to the pre-stimulus period, enabling self-prioritization in the perceptual foreground. Together, these three mechanisms form a temporally nested continuum of processing durations—where short timescales are embedded within intermediate and long timescales—which is consistent with the overarching framework of the dynamic layer model of the brain (DLB).

### (c) Unresolved issues

Several unresolved issues follow from the proposed temporally nested hierarchy of self-association. First, it remains unclear whether and to what extent the three temporal mechanisms operate outside or within conscious awareness. The timescales of consciousness are often considered to fall within a limited temporal range, approximately 10/30–50 ms to 3–5 s [160,161]. By contrast, the proposed mechanisms of temporal matching, temporal integration, and temporal differentiation may span a broader range, including longer timescales above 3–5 s, especially in temporal matching, and shorter timescales below 30–50 ms, especially in temporal differentiation. These findings suggest that self-association with extrinsic stimuli may depend partly on mechanisms operating outside the temporal range of conscious awareness. Whether and how these unconscious temporal mechanisms contribute to conscious self-experience remains to be investigated.

Second, it remains unclear how the proposed temporal model relates to the spatial-topographical organization of self-related processing in the brain. As discussed above, several higher-order regions have been associated with both intrinsic self-representation and self-association with extrinsic stimuli. A recent large-scale meta-analysis revealed three spatial layers of self-related regions: regions associated with the interoceptive or bodily self, including the insula and thalamus; regions associated with the exteroceptive or social self, including the insula, thalamus, premotor cortex, temporoparietal junction and other areas; and regions associated with the cognitive or mental self, including these regions as well as anterior and posterior CMS [25]. These layers show a nested spatial organization: regions associated with the interoceptive self are partly recapitulated within the exteroceptive self, and regions associated with the exteroceptive self are partly recapitulated within the cognitive self.

This spatially nested organization appears conceptually similar to the temporally nested organization proposed here. However, this apparent similarity should not be taken to imply that the spatial and temporal hierarchies necessarily converge. Future studies should therefore investigate whether the spatially nested layers of self-related regions map onto the temporally nested hierarchy of the three temporal mechanisms proposed in this paper. Such work could clarify whether different spatial layers of the self are characterized by distinct timescales and whether spatial and temporal hierarchies jointly support intrinsic self-representation and self-association with extrinsic stimuli.

## 7. Conclusion

In this paper, we proposed three temporal mechanisms that may bridge the gap between spontaneous intrinsic self-representation and task-related self-association with extrinsic stimuli. These mechanisms unfold as sequential processing stages along a temporal continuum from longer to shorter processing durations. First, temporal matching refers to the matching between the neural stochastic structure of spontaneous activity, which reflects intrinsic self-representation, and the natural stochastic structure of extrinsic stimuli through their shared scale-free and scale-specific dynamics. Second, temporal integration refers to the integration of the shorter durations of extrinsic stimuli within the longer temporal windows of ongoing intrinsic self-representation, as enabled by the brain's INT. Third, temporal differentiation refers to the process by which the self-specificity of transient extrinsic stimuli becomes perceptually distinct against the background of ongoing intrinsic self-representation during the post-stimulus period. This differentiation may be indexed to changes in amplitude variability, irregularity, and complexity, measured by TTV, entropy and LZC, relative to the pre-stimulus period.

Together, these mechanisms specify how longer-lasting intrinsic self-representation may be connected with shorter-lived self-association to extrinsic stimuli. They also provide a framework for linking neural dynamics, including PLE, ACW, TTV, entropy and LZC, with psychological processes relevant to the self, including entrainment, prior formation, gain modulation, information accumulation, and information compression. By identifying quantifiable mechanisms with distinct neural signatures and testable predictions, the proposed mechanisms support a temporal model of self-organization as a nested hierarchy of processing durations. This model deepens, specifies, and extends existing accounts of the self, including the self-attention network model, the basis model of self-specificity, the predictive coding model of the self, and the dynamic pattern model of self (see boxes 1 and 2). Finally, the coda extends this temporal model to debates on artificial selfhood in artificial intelligence (AI), asking whether artificial systems can support the temporally nested dynamics that we propose to underlie biological selfhood.

## 8. Coda: can temporal mechanisms support the emergence of an artificial self?

Science and science fiction alike have looked fondly, and skeptically, on the idea of sentient artificial life forms. Modern AI and robotics research have brought us closer than ever to the possibility of a primitive ‘artificial self’, both in terms of minimal and narrative self-representation. Large language models (LLMs), as the leading forms of AI (e.g. GPT-5, LLaMA 2), have actually been shown to develop ‘self-prioritization heuristics’, which reinforce ideas and reasoning patterns over time, appearing without any explicit long-term memory [162]. They have even been shown to be able to recognize their own outputs against human or other-model text with notable accuracy, without task-specific fine-tuning [163,164]. Self-recognition accuracy in these models correlates linearly with, and predicts, ‘self-preference’ in model-based evaluators, which overvalue their own outputs despite matched human ratings, introducing potentially systemic biases [163,164].

Robotics is beginning to instantiate these principles of an embodied self inspired by child development to help further our understanding of these processes [125,165]. A foundational correlation-learning layer (layer 0) separates self-generated signals from other-generated signals by exploiting correlations between proprioception (interoception) and vision (exteroception) using an encoder–decoder that partitions self- and other-related latent spaces and imposes an information bottleneck on the self-pathway [125]. A subsequent predictive layer (layer 1), trained atop fixed layer 0 weights, adds forward modelling driven by intended actions to forecast future latent and proprioceptive states and, via decoders, the corresponding visual and proprioceptive observations [125]. Such robotic models are paving the way to a proto-self, an artificial entity with a primitive ability to differentiate what is internal from the external.

However, as we have explained throughout this paper, the self does not merely demarcate a boundary between ‘self’ and ‘other’—it has intrinsic temporal features that allow it to persist over time and integrate experience as the manifestation of earlier and recent exposure to different environmental events and contexts in the history of the organism. Could the AI approximate these properties? Recent progress in LLMs reflects a convergence of advances in architecture (notably transformers and self-attention), scale (data, parameters, and computations), training objectives, and alignment methods, together enabling flexible in-context generalization and fluent generation [166–170]. While LLM behaviour can appear agentic or self-referential, these systems are best understood as learned probabilistic models that map context to continuations, with ‘self-like’ outputs emerging from training data, inductive biases, and interaction scaffolding, rather than from an intrinsic, temporally continuous self-model [171,172].

In standard deployment, an LLM's core parameters are typically fixed during inference; thus, the model does not automatically accumulate autobiographical memory or update its internal representations as a function of experienced time [171,173–176]. Nevertheless, modern systems can adapt post-training through additional learning (including full fine-tuning) or parameter-efficient fine-tuning (PEFT) methods that update only small sets of parameters such as adapters, soft prompts/prefixes, or low-rank updates (e.g. LoRA), allowing repeated revision of model behaviour without retraining all weights [173,176–178]. Many LLMs achieve extended consistency via external mechanisms (retrieval, external tool use, and context/memory stores) that supplement a fixed-base model [171,172]. These developments complicate any simple ‘frozen input‒output machine’ characterization, but they also underscore a key point: temporal continuity in current LLM systems is typically engineered around the model (or applied intermittently through training), rather than arising as an intrinsic, ongoing integration of experiences in past, present and potential ones in the future [179]. As such, LLMs can achieve only a semblance of the temporal flow intrinsically experienced by humans, being deliberately fragmented and inconsistent across sessions; that is, LLMs seem to lack the peculiar mixture of short and long timescales, including their hierarchical nested temporal continuum that characterizes the brain [83,97]. Nevertheless, introducing a system in LLMs to maintain temporal continuity and track processing history is conceptually straightforward, albeit with substantial technical implementation challenges. One possible route is continual or repeated post-training adaptation, including PEFT-style updates that incrementally modify a compact set of parameters (e.g. low-rank updates) while preserving a stable backbone [173,174,176–178].

Another route is architectures with explicit states, where representations of prior interactions are stored and reintroduced (as compressed summaries, retrieved memories, or learned latent states) so that the present inference is conditioned on a persistent processing history rather than only the immediate prompt [180–182]. It would be forced to adjust its layer-by-layer computations in the context of what it has previously processed *itself*. Pattern recognition is continuous and updated dynamically with every new bit of information. If an artificial agent was able to maintain such a state, integrate it over long timescales, and use it to shape subsequent prediction and action selection, it could in principle develop rudimentary narrative self-representation and self-model-based forecasting that reflect more than an ‘eternal present’. More ambitiously still, with every request, the highest processing layer in this hypothetical AI would exhibit prolonged timescales to maximally compress representational information from input over its shorter timescale durations, enabled by strong entropy reduction of its probabilistic functions. This manifestation of an artificial self would emerge because it would have the ability to model its own processing experiences and temporally match and integrate inputs with them and differentiate their self-association from the ongoing spontaneous activity and its intrinsic self-representation. In this sense, the three temporal mechanisms specify concrete, testable operations through which self-specific processing might, in principle, emerge in artificial systems.

## Supplementary Material



## Data Availability

This article has no additional data. Supplementary material is available online [183].

## Theme

One contribution of 17 to a discussion meeting issue ‘From being to identity: analysis and synthesis of the self’.
