# Supplementary material for: From rest to task: temporal mechanisms of self-association
Source: Philos Trans R Soc Lond B Biol Sci. 2026 Nov 10;381(1962):20250346. doi: 10.1098/rstb.2025.0346 (PMC13750416; doi:10.1098/rstb.2025.0346)
Supplement: Supplementary file 1 [file rstb.2025.0346.zip › rstb.2025.0346/rstb20250346_si_001.docx]

# Supplementary Material

All evidence in the Supplementary Material is summarized in Table 1 in the main text.

## Part I: Evidence of Self-Referential Processing from Task-Related Activity

## Self-specificity enhances late processing in the P3

Self-referential processing has been widely used to study the effects of the self on task-related activity, particularly in EEG. One of the most reliable results across different experimental paradigms is the observed effect in the P3 event-related potential (ERP) occurring 300-500ms after stimulus onset. Across different experimental paradigms, P3 amplitude has been shown to be increased in response to self-specific stimuli compared to non-self-specific stimuli. Importantly, this enhancement occurs across a variety of different stimuli including one’s own name, face, handwriting, car, emotions, source memory, ambiguity, hometown, and national flag (Berlad & Pratt, 1995; A. Chen et al., 2008; D. Chen et al., 2024; Deng et al., 2022; Fan et al., 2011; Folmer & Yingling, 1997; Gray et al., 2004; Katyal et al., 2020; Kotlewska et al., 2023; Li et al., 2022; Ma & Han, 2010; Nie et al., 2023; Porter et al., 2021; Sui et al., 2009, 2023; Tacikowski & Nowicka, 2010). This suggests domain-independence of the P3 effect in self-specific tasks, meaning that self-specificity is the driving factor regardless of the modality, or domain, of the stimuli (verbal, non-verbal, emotional, etc.). The domain-independence of P3 as a temporal effect (300-500ms after stimulus onset) converges spatially with similar self-specific domain-independence observed in the cortical midline structures, which form the core of the DMN (Frewen et al., 2020; Northoff et al., 2006; van der Meer et al., 2010).

Is the augmentation of P3 amplitude simply an effect of the familiarity of the self-specific stimuli? A recent EEG study addressed this by controlling for familiarity of the stimuli, presenting self-specific (self), familiar (friend), and non-familiar (stranger) stimuli in a self-prioritization task (Sui et al., 2023). In this task, participants learned shape-label associations for the three identities, and in accordance with previous results, they observed a significant increase in P3 amplitude for self-specific stimuli, in comparison to familiar and unfamiliar conditions. Being that in mismatching trials P3 amplitude was not increased, it is the association of the shape with the self, not just the shape, that modulates this effect. Moreover, Sui and colleagues (Sui et al., 2023) also found P3 amplitude significantly impacted the psychological decision threshold, estimated with hierarchical drift diffusion modelling (HDDM). High P3 amplitudes were significantly related to a narrower threshold, indicating less evidence is required to reach a decision when stimuli are self-specific.

### Self-specificity enhances early processing in N1

In addition to the late P3 ERP, there is evidence for the involvement of earlier ERPs, N1/N2, in self-specific processing. Studies have shown decreased N170/N2 when processing one’s own face, suggested to be related to the decreased level of surprise when processing such stimuli (Bola et al., 2021; Keyes et al., 2010; Sui et al., 2006; Sui, Hong, et al., 2013; Żochowska & Nowicka, 2024). However, when controlling for familiarity in self-processing, Wozniak et al. (Woźniak et al., 2018) found the self-related N2 decrease persisted, implying that this effect is not due to familiarity but to the association of the self with the stimulus (Woźniak et al., 2018; Żochowska & Nowicka, 2024). Furthermore, the N1 is modulated when words are associated with self-relevant linguistic contexts (Fields & Kuperberg, 2012), moral contexts (Zhan et al., 2020), and when self-faces are used as endogenous cues (Liu et al., 2016). Finally, Pinheiro and colleagues (Pinheiro et al., 2023) showed early amplitude changes in N1 and P2 during self-voice perception which was modulated by both attention and emotion, implying early automatic processing of self-reference.

Evidence for the involvement of early N1 in self-referential processing is presented by Sui et al. (Sui et al., 2023), who examined this component in addition to P3. They found its negative amplitude was significantly stronger for self-matching stimuli in comparison to friend or stranger matching trials. Reflecting self-prioritization, this difference was not significant for mismatching trials, demonstrating that N1 processing relates to the successful *association* of the self with the shape. Moreover, N1 amplitude significantly modulated drift rates in HDDM, where a greater, negative amplitude was related to faster drift rates, indicating faster, more accurate responding.

### Top-down feedback processing and early alpha-based dynamic functional connectivity

What might be the neural basis of this top-down modulation by the intrinsic self-representation? Lu and colleagues (Lu et al., 2024) used EEG during the same self-matching task to investigate fronto-occipital functional connectivity (FC) in the alpha band (8–12Hz). They did so in a dynamic way, by examining FC at each sampling point from prestimulus (-200–0ms) to poststimulus (0–100ms). They observed three main temporal clusters of alpha fronto-occipital FC. The first cluster showed relatively weak FC directed from frontal to occipital electrodes during the prestimulus period (-200–0ms) (see below for more details on prestimulus activity modulating self-specific processing). Next, after stimulus onset, FC from frontal to occipital electrodes increased, peaking around 150–180ms — this was significantly stronger in the self-matching condition compared to both friend and stranger condition. Importantly, the same increase in frontal-to-occipital FC was not observed in the mismatching conditions, including self-mismatch (Kotlewska et al., 2023; Lu et al., 2024). We can tentatively conclude that the intrinsic self-representation may modulate the processing of extrinsic self-labelled stimuli, supporting the assumption that early top-down feedback modulation of fronto-occipital FC is related to this association. Therefore, the results are promising for the implication of the intrinsic self-representation in early entry effects (Constable et al., 2019; Jublie & Kumar, 2021; Truong et al., 2017). Further topographic evidence for the key role of frontal regions in such top-down modulation comes from fMRI studies, which show that self-prioritization is associated with mainly prefrontal regions like the default-mode network (Frewen et al., 2020; Northoff et al., 2006; Qin et al., 2020; Sui et al., 2015; Sui, Rotshtein, et al., 2013). This aligns well with the findings by Lu et al. (Lu et al., 2024) discussed here.

Moreover, FC was also examined in the opposite direction, from occipital to frontal electrodes (Lu et al., 2024). The authors found evidence of later FC 229-272ms to 670-870ms poststimulus across all conditions. However, self-matching trials exhibited significantly lower occipito-frontal FC than both stranger and friend matching trials. These findings suggest weaker bottom-up feedforward modulation from occipital to frontal electrodes, which indicates that in comparison to non-self-specific stimuli, there is less sensory processing of self-specific stimuli. These results complement the stronger top-down fronto-occipital FC in self-related processing, with greater modulation by frontal areas and less need for bottom-up sensory processing, given that alpha FC is known to be related to input processing (Klimesch, 2012). This may also be computationally framed as the balance between prior, early top-down feedback connections, and posterior, late bottom-up feedforward connections (Scheller, Fang, et al., 2024; Scheller & Sui, 2022).

Recent research has demonstrated that, in addition to the alpha band (8–12Hz) which has been consistently linked to self-referential processing (Knyazev, 2013), other EEG frequency bands are also involved. For instance, studies have highlighted the role of the gamma band (30–80Hz), particularly 40Hz frequencies, in mediating self-referential processes (Bocharov et al., 2019; Kraus et al., 2021; Lou et al., 2010, 2017). Moreover, emerging evidence suggests that the beta band (13–30 Hz) may contribute to self-referential processing as well, potentially by facilitating motor-related processes that result in faster reaction times (Alzueta et al., 2020; Haciahmet et al., 2023). A recent study associated beta activity with the evidence accumulation (HDDM) during self-prioritization of external stimuli (Haciahmet et al., 2023). Additional studies have also implicated the delta and theta bands in self-referential activity (Haciahmet et al., 2023; Kotlewska et al., 2023), along with further support for the gamma band across several investigations (Lou et al., 2010, 2017, 2020). Overall, these findings suggest that nearly all canonical EEG frequency bands play a role in self-referential processing. This broad involvement implies a potentially global temporal neural mechanism, where multiple frequency bands may interact to support self-related cognition (Mu & Han, 2010). As we will explore further below, there is growing empirical evidence supporting such global-temporal, integrative, cross-frequency dynamics in self-referential processing.

## Part II: Spontaneous Activity and the Intrinsic Self-Representation

There is abundant evidence of the involvement of an intrinsic self-representation in stimulus-related activity, but where does this representation come from? If it is intrinsic, it must be stored prior to extrinsic input processing. Recent psychological and computational studies suggest that there is indeed a prior, the self, that modulates the processing of the posterior, or extrinsic stimuli (Scheller, Tünnermann, et al., 2024; Scheller & Sui, 2022). Various fMRI studies show that resting-state activity in the CMS, the core regions of the DMN (Andrews‐Hanna et al., 2014), strongly overlaps with task-related activity in the same regions during self-specific processing (D’Argembeau et al., 2005; Davey et al., 2016; Frewen et al., 2020; Knyazev, 2013; Qin et al., 2020; Qin & Northoff, 2011; Schneider et al., 2008; Sui et al., 2023; Sui, Rotshtein, et al., 2013; Whitfield-Gabrieli et al., 2011). Other studies show electrophysiological overlap using intra-cranial recordings, finding that firing rates in the anterior cingulate cortex (ACC) did not change from rest to task while processing self-specific stimuli, but did change for non-self-specific stimuli (Dastjerdi et al., 2011; Lipsman et al., 2014; Tan et al., 2022). Such rest-self overlap suggests, indirectly, that the brain’s spontaneous activity in the CMS/DMN may be an important component in the intrinsic self-representation. The relationship between spontaneous and task-related self-specific activity within these regions is yet to be uncovered, therefore we now discuss the temporal features of spontaneous activity in hopes of discerning this relationship.

### Prestimulus Modulation of Self-Specificity

Evidence of an intrinsic self-representation comes from studies looking at how prestimulus, spontaneous activity is implicated in self-specific processing. First, we discuss results from fMRI. Schneider and colleagues (Schneider et al., 2008) conducted an fMRI study where participants had to judge the degree of self-specificity of emotional and neutral stimuli whose ratings were then correlated with the activity in the prestimulus interval preceding the rated stimulus. They observed that prestimulus activity in the ventromedial prefrontal cortex (vmPFC) and the posterior cingulate cortex (PCC) of the CMS was directly related to the participants’ ratings of stimulus self-specificity. The higher the self-specificity, the higher the intertrial activity in the vmPFC and the PCC. Notably, activity levels in these regions could be distinguished from the activity related to emotional vs. neutral ratings and the stimuli themselves (Northoff et al., 2009; Schneider et al., 2008).

Furthermore, Qin et al. (Qin et al., 2016) used fMRI to investigate self vs. unknown name perception by embedding these stimuli in noise. They found that identification of self-name within the noise was predicted by stronger prestimulus CMS activity, as well as stronger activity in temporal regions like the temporo-parietal junction (TPJ) and the temporal pole. This is in line with Meyer and Lieberman’s findings (Meyer & Lieberman, 2018), who observed that higher fMRI activity in intertrial periods related to self-referential thinking in stimulus processing, while weaker activity was related to other-referential thinking.

Analogous to these fMRI findings, EEG studies have also found significant prestimulus effects on task-related self processing. Bai et al. (Bai et al., 2016) observed that higher alpha power in prestimulus intervals predicted a higher degree of self-relatedness assigned to stimuli. Together, both fMRI and EEG studies suggest that the prestimulus period, reflecting the brain’s spontaneous activity, contains some baseline information about self-specificity. This may reflect the manifestation of the supposed intrinsic self-representation. Interestingly, for self-referential processing, the role of alpha power in prestimulus activity converges with its role in poststimulus activity (Kraus et al., 2021; Lu et al., 2024). Tentatively, one may assume that alpha may be the key to self-specificity by carrying over this information from pre to poststimulus activity. This is firmly in line with the assumption that alpha operates as a gate for extrinsic input/stimulus processing (Klimesch, 2012).

### Resting-State Temporal Dynamics and the Intrinsic Self-Representation

Moving beyond static measures of neural activity, we now consider the dynamic, temporal features of the intrinsic self-representation. First, Huang and collaborators (Huang et al., 2016) investigated the overall shape of the infraslow power spectrum (0.01–0.1Hz) using resting-state fMRI. They computed the power law exponent (PLE) on the infraslow spectrum to investigate the relationship between resting-state dynamics and participants’ scores on the Self-Consciousness Scale (SCS) in private, public, and social dimensions. The PLE describes the slope of the power spectrum, which reflects the balance in the power of slower and faster frequencies. If power is stronger in the slower frequencies relative to the faster frequencies, the slope is steep, yielding high PLE values. If the reverse is true, PLE values are low. In this way, PLE measures the degree to which faster frequency power is embedded within the much stronger power of slow frequencies within the power spectrum. Their findings show a direct relationship of the PLE in CMS with self-consciousness: the higher participants’ PLE, the higher their scores on the private self-consciousness scale. In contrast, such a correlation was not observed for public or social dimensions of the SCS. This suggests that private self-consciousness has an intimate relationship with the power balance of faster and slower frequencies in the infraslow domain in fMRI.

Would an analogous dynamic relationship also hold for faster frequency bands? This was investigated using EEG by Wolff et al. (Wolff et al., 2019), who confirmed results in (Huang et al., 2016), finding a significant correlation of private SCS scores with resting-state PLE in the 1-50Hz range. The higher the PLE, the higher the private SCS scores, a correlation which was not significant for public or social SCS dimensions. Notably, power in individual frequency bands did not correlate with any SCS scores. Finally, they showed that the relationship of private SCS with the PLE is especially strong in the vmPFC and PCC regions of the CMS. In essence, these resting-state fMRI and EEG findings support that it is the dynamics of spontaneous activity across frequencies that encodes an intrinsic self-representation.

Moreover, the dynamics of neural activity can also be investigated in terms of its timescales. These intrinsic neural timescales (INTs) represent the duration of a neuronal process for both resting-state and task-related activity (Hasson et al., 2015; Wolff et al., 2022). INTs are computed with the autocorrelation function, measuring the degree to which a signal correlates with itself over time (Golesorkhi, Gomez-Pilar, Tumati, et al., 2021; Golesorkhi, Gomez-Pilar, Zilio, et al., 2021; Honey et al., 2012; Murray et al., 2014). The longer the autocorrelation, the longer the autocorrelation window (ACW) and thus the longer the duration of the ongoing neural process. Wolff et al. (Wolff et al., 2019) demonstrated that longer ACW in resting-state led to higher private SCS scores. This highlights the role of the brain’s INTs in mediating the intrinsic self-representation within the brain’s spontaneous activity. Other studies have similarly documented the tight link between the degree of self-identity and the strength of long-range temporal correlations, as measured with detrended fluctuation analysis (DFA) (Hardstone et al., 2012; Linkenkaer-Hansen et al., 2001), where stronger temporal correlations predicted stronger self-identity (Sugimura et al., 2021). In summary, the dynamic structure of spontaneous activity as measured with PLE, ACW and other methods seems to be critical to encoding of an intrinsic self-representation.

# References

Alzueta, E., Melcón, M., Jensen, O., & Capilla, A. (2020). The “Narcissus Effect”: Top-down alpha-beta band modulation of face-related brain areas during self-face processing. *NeuroImage*, *213*, 116754. https://doi.org/10.1016/j.neuroimage.2020.116754

Andrews‐Hanna, J. R., Smallwood, J., & Spreng, R. N. (2014). The default network and self‐generated thought: Component processes, dynamic control, and clinical relevance. *Annals of the New York Academy of Sciences*, *1316*(1), 29–52. https://doi.org/10.1111/nyas.12360

Bai, Y., Nakao, T., Xu, J., Qin, P., Chaves, P., Heinzel, A., Duncan, N., Lane, T., Yen, N.-S., Tsai, S.-Y., & Northoff, G. (2016). Resting state glutamate predicts elevated pre-stimulus alpha during self-relatedness: A combined EEG-MRS study on “rest-self overlap.” *Social Neuroscience*, *11*(3), 249–263. https://doi.org/10.1080/17470919.2015.1072582

Berlad, I., & Pratt, H. (1995). P300 in response to the subject’s own name. *Electroencephalography and Clinical Neurophysiology/Evoked Potentials Section*, *96*(5), 472–474. https://doi.org/10.1016/0168-5597(95)00116-A

Bocharov, A. V., Knyazev, G. G., Savostyanov, A. N., Astakhova, T. N., & Tamozhnikov, S. S. (2019). EEG dynamics of spontaneous stimulus-independent thoughts. *Cognitive Neuroscience*, *10*(2), 77–87. https://doi.org/10.1080/17588928.2018.1534820

Bola, M., Paź, M., Doradzińska, Ł., & Nowicka, A. (2021). The self-face captures attention without consciousness: Evidence from the N2pc ERP component analysis. *Psychophysiology*, *58*(4), e13759. https://doi.org/10.1111/psyp.13759

Chen, A., Weng, X., Yuan, J., Lei, X., Qiu, J., Yao, D., & Li, H. (2008). The Temporal Features of Self-referential Processing Evoked by Chinese Handwriting. *Journal of Cognitive Neuroscience*, *20*(5), 816–827. https://doi.org/10.1162/jocn.2008.20505

Chen, D., Liu, Y., Zhang, Y., & Guo, Y. (2024). The neural basis of self-ambivalence: An ERP study. *BMC Psychology*, *12*(1), 758. https://doi.org/10.1186/s40359-024-02257-9

Constable, M. D., Welsh, T. N., Huffman, G., & Pratt, J. (2019). I before U: Temporal order judgements reveal bias for self-owned objects. *Quarterly Journal of Experimental Psychology*, *72*(3), 589–598. https://doi.org/10.1177/1747021818762010

D’Argembeau, A., Collette, F., Van der Linden, M., Laureys, S., Del Fiore, G., Degueldre, C., Luxen, A., & Salmon, E. (2005). Self-referential reflective activity and its relationship with rest: A PET study. *Neuroimage*, *25*(2), 616–624.

Dastjerdi, M., Foster, B. L., Nasrullah, S., Rauschecker, A. M., Dougherty, R. F., Townsend, J. D., Chang, C., Greicius, M. D., Menon, V., Kennedy, D. P., & Parvizi, J. (2011). Differential electrophysiological response during rest, self-referential, and non-self-referential tasks in human posteromedial cortex. *Proceedings of the National Academy of Sciences of the United States of America*, *108*(7), 3023–3028. https://doi.org/10.1073/pnas.1017098108

Davey, C. G., Pujol, J., & Harrison, B. J. (2016). Mapping the self in the brain’s default mode network. *NeuroImage*, *132*, 390–397.

Deng, N., Sun, Y., Chen, X., & Li, W. (2022). How does self name influence the neural processing of emotional prosody? An ERP study. *PsyCh Journal*, *11*(1), 30–42. https://doi.org/10.1002/pchj.499

Fan, W., Zhang, Y., Wang, X., Wang, X., Zhang, X., & Zhong, Y. (2011). The temporal features of self-referential processing evoked by national flag. *Neuroscience Letters*, *505*(3), 233–237. https://doi.org/10.1016/j.neulet.2011.10.017

Fields, E. C., & Kuperberg, G. R. (2012). It’s all about you: An ERP study of emotion and self-relevance in discourse. *NeuroImage*, *62*(1), 562–574. https://doi.org/10.1016/j.neuroimage.2012.05.003

Folmer, R. L., & Yingling, C. D. (1997). Auditory P3 Responses to Name Stimuli. *Brain and Language*, *56*(2), 306–311. https://doi.org/10.1006/brln.1997.1828

Frewen, P., Schroeter, M. L., Riva, G., Cipresso, P., Fairfield, B., Padulo, C., Kemp, A. H., Palaniyappan, L., Owolabi, M., & Kusi-Mensah, K. (2020). Neuroimaging the consciousness of self: Review, and conceptual-methodological framework. *Neuroscience & Biobehavioral Reviews*, *112*, 164–212.

Golesorkhi, M., Gomez-Pilar, J., Tumati, S., Fraser, M., & Northoff, G. (2021). Temporal hierarchy of intrinsic neural timescales converges with spatial core-periphery organization. *Communications Biology*, *4*(1), Article 1. https://doi.org/10.1038/s42003-021-01785-z

Golesorkhi, M., Gomez-Pilar, J., Zilio, F., Berberian, N., Wolff, A., Yagoub, M. C. E., & Northoff, G. (2021). The brain and its time: Intrinsic neural timescales are key for input processing. *Communications Biology*, *4*, 970. https://doi.org/10.1038/s42003-021-02483-6

Gray, H. M., Ambady, N., Lowenthal, W. T., & Deldin, P. (2004). P300 as an index of attention to self-relevant stimuli. *Journal of Experimental Social Psychology*, *40*(2), 216–224. https://doi.org/10.1016/S0022-1031(03)00092-1

Haciahmet, C. C., Golubickis, M., Schäfer, S., Frings, C., & Pastötter, B. (2023). The oscillatory fingerprints of self-prioritization: Novel markers in spectral EEG for self-relevant processing. *Psychophysiology*, *60*(12), e14396. https://doi.org/10.1111/psyp.14396

Hardstone, R., Poil, S.-S., Schiavone, G., Jansen, R., Nikulin, V. V., Mansvelder, H. D., & Linkenkaer-Hansen, K. (2012). Detrended fluctuation analysis: A scale-free view on neuronal oscillations. *Frontiers in Physiology*, *3*, 450. https://doi.org/10.3389/fphys.2012.00450

Hasson, U., Chen, J., & Honey, C. J. (2015). Hierarchical process memory: Memory as an integral component of information processing. *Trends in Cognitive Sciences*, *19*(6), 304–313. https://doi.org/10.1016/j.tics.2015.04.006

Honey, C. J., Thesen, T., Donner, T. H., Silbert, L. J., Carlson, C. E., Devinsky, O., Doyle, W. K., Rubin, N., Heeger, D. J., & Hasson, U. (2012). Slow cortical dynamics and the accumulation of information over long timescales. *Neuron*, *76*(2), 423–434. https://doi.org/10.1016/j.neuron.2012.08.011

Huang, Z., Obara, N., Davis, H. (Hap), Pokorny, J., & Northoff, G. (2016). The temporal structure of resting-state brain activity in the medial prefrontal cortex predicts self-consciousness. *Neuropsychologia*, *82*, 161–170. https://doi.org/10.1016/j.neuropsychologia.2016.01.025

Jublie, A., & Kumar, D. (2021). Early Capture of Attention by Self-Face: Investigation Using a Temporal Order Judgment Task. *I-Perception*, *12*(4), 20416695211032993. https://doi.org/10.1177/20416695211032993

Katyal, S., Hajcak, G., Flora, T., Bartlett, A., & Goldin, P. (2020). Event-related potential and behavioural differences in affective self-referential processing in long-term meditators versus controls. *Cognitive, Affective & Behavioral Neuroscience*, *20*(2), 326–339. https://doi.org/10.3758/s13415-020-00771-y

Keyes, H., Brady, N., Reilly, R. B., & Foxe, J. J. (2010). My face or yours? Event-related potential correlates of self-face processing. *Brain and Cognition*, *72*(2), 244–254.

Klimesch, W. (2012). α-band oscillations, attention, and controlled access to stored information. *Trends in Cognitive Sciences*, *16*(12), 606–617. https://doi.org/10.1016/j.tics.2012.10.007

Knyazev, G. G. (2013). EEG Correlates of Self-Referential Processing. *Frontiers in Human Neuroscience*. https://doi.org/10.3389/fnhum.2013.00264

Kotlewska, I., Panek, B., Nowicka, A., & Asanowicz, D. (2023). Posterior theta activity reveals an early signal of self-face recognition. *Scientific Reports*, *13*(1), 13823. https://doi.org/10.1038/s41598-023-41071-y

Kraus, B., Salvador, C. E., Kamikubo, A., Hsiao, N.-C., Hu, J.-F., Karasawa, M., & Kitayama, S. (2021). Oscillatory alpha power at rest reveals an independent self: A cross-cultural investigation. *Biological Psychology*, *163*, 108118.

Li, B., Hu, W., Hunt, A., & Sui, J. (2022). Self-related objects increase alertness and orient attention through top-down saliency. *Attention, Perception, & Psychophysics*, *84*(2), 408–417. https://doi.org/10.3758/s13414-021-02429-4

Linkenkaer-Hansen, K., Nikouline, V. V., Palva, J. M., & Ilmoniemi, R. J. (2001). Long-range temporal correlations and scaling behavior in human brain oscillations. *Journal of Neuroscience*, *21*(4), 1370–1377.

Lipsman, N., Nakao, T., Kanayama, N., Krauss, J. K., Anderson, A., Giacobbe, P., Hamani, C., Hutchison, W. D., Dostrovsky, J. O., Womelsdorf, T., Lozano, A. M., & Northoff, G. (2014). Neural overlap between resting state and self-relevant activity in human subcallosal cingulate cortex—Single unit recording in an intracranial study. *Cortex; a Journal Devoted to the Study of the Nervous System and Behavior*, *60*, 139–144. https://doi.org/10.1016/j.cortex.2014.09.008

Liu, M., He, X., Rotsthein, P., & Sui, J. (2016). Dynamically orienting your own face facilitates the automatic attraction of attention. *Cognitive Neuroscience*, *7*(1–4), 37–44. https://doi.org/10.1080/17588928.2015.1044428

Lou, H. C., Changeux, J. P., & Rosenstand, A. (2017). Towards a cognitive neuroscience of self-awareness. *Neuroscience and Biobehavioral Reviews*, *83*, 765–773. https://doi.org/10.1016/j.neubiorev.2016.04.004

Lou, H. C., Gross, J., Biermann-Ruben, K., Kjaer, T. W., & Schnitzler, A. (2010). Coherence in consciousness: Paralimbic gamma synchrony of self-reference links conscious experiences. *Human Brain Mapping*, *31*(2), 185–192. https://doi.org/10.1002/hbm.20855

Lou, H. C., Rømer Thomsen, K., & Changeux, J.-P. (2020). The Molecular Organization of Self-awareness: Paralimbic Dopamine-GABA Interaction. *Frontiers in Systems Neuroscience*, *14*. https://doi.org/10.3389/fnsys.2020.00003

Lu, Z., He, X., Yi, D., & Sui, J. (2024). Temporal properties of self-prioritization. *Entropy*, *26*(3), 242.

Ma, Y., & Han, S. (2010). Why we respond faster to the self than to others? An implicit positive association theory of self-advantage during implicit face recognition. *Journal of Experimental Psychology: Human Perception and Performance*, *36*(3), 619–633. https://doi.org/10.1037/a0015797

Meyer, M. L., & Lieberman, M. D. (2018). Why people are always thinking about themselves: Medial prefrontal cortex activity during rest primes self-referential processing. *Journal of Cognitive Neuroscience*, *30*(5), 714–721.

Mu, Y., & Han, S. (2010). Neural oscillations involved in self-referential processing. *NeuroImage*, *53*(2), 757–768. https://doi.org/10.1016/j.neuroimage.2010.07.008

Murray, J. D., Bernacchia, A., Freedman, D. J., Romo, R., Wallis, J. D., Cai, X., Padoa-Schioppa, C., Pasternak, T., Seo, H., Lee, D., & Wang, X.-J. (2014). A hierarchy of intrinsic timescales across primate cortex. *Nature Neuroscience*, *17*(12), 1661–1663. https://doi.org/10.1038/nn.3862

Nie, A., Zhou, W., & Xiao, Y. (2023). Sensitivity of late ERP old/new effects in source memory to self-referential encoding focus and stimulus emotionality. *Neurobiology of Learning and Memory*, *203*, 107795. https://doi.org/10.1016/j.nlm.2023.107795

Northoff, G., Heinzel, A., De Greck, M., Bermpohl, F., Dobrowolny, H., & Panksepp, J. (2006). Self-referential processing in our brain—A meta-analysis of imaging studies on the self. *Neuroimage*, *31*(1), 440–457.

Northoff, G., Schneider, F., Rotte, M., Matthiae, C., Tempelmann, C., Wiebking, C., Bermpohl, F., Heinzel, A., Danos, P., Heinze, H.-J., Bogerts, B., Walter, M., & Panksepp, J. (2009). Differential parametric modulation of self-relatedness and emotions in different brain regions. *Human Brain Mapping*, *30*(2), 369–382. https://doi.org/10.1002/hbm.20510

Pinheiro, A. P., Sarzedas, J., Roberto, M. S., & Kotz, S. A. (2023). Attention and emotion shape self-voice prioritization in speech processing. *Cortex*, *158*, 83–95.

Porter, N. A., Fields, E. C., Moore, I. L., & Gutchess, A. (2021). Late frontal positivity effects in Self-referential Memory: Unique to the Self? *Social Neuroscience*, *16*(4), 406–422. https://doi.org/10.1080/17470919.2021.1929460

Qin, P., Grimm, S., Duncan, N. W., Fan, Y., Huang, Z., Lane, T., Weng, X., Bajbouj, M., & Northoff, G. (2016). Spontaneous activity in default-mode network predicts ascription of self-relatedness to stimuli. *Social Cognitive and Affective Neuroscience*, *11*(4), 693–702.

Qin, P., & Northoff, G. (2011). How is our self related to midline regions and the default-mode network? *NeuroImage*. https://doi.org/10.1016/j.neuroimage.2011.05.028

Qin, P., Wang, M., & Northoff, G. (2020). Linking bodily, environmental and mental states in the self—A three-level model based on a meta-analysis. *Neuroscience & Biobehavioral Reviews*, *115*, 77–95. https://doi.org/10.1016/j.neubiorev.2020.05.004

Scheller, M., Fang, H., & Sui, J. (2024). Self as a prior: The malleability of Bayesian multisensory integration to social salience. *British Journal of Psychology*, *115*(2), 185–205. https://doi.org/10.1111/bjop.12683

Scheller, M., & Sui, J. (2022). The power of the self: Anchoring information processing across contexts. *Journal of Experimental Psychology: Human Perception and Performance*, *48*(9), 1001–1021. https://doi.org/10.1037/xhp0001017

Scheller, M., Tünnermann, J., Fredriksson, K., Fang, H., & Sui, J. (2024). Self-association enhances early attentional selection through automatic prioritization of socially salient signals. *eLife*, *13*. https://doi.org/10.7554/eLife.100932.2

Schneider, F., Bermpohl, F., Heinzel, A., Rotte, M., Walter, M., Tempelmann, C., Wiebking, C., Dobrowolny, H., Heinze, H. J., & Northoff, G. (2008). The resting brain and our self: Self-relatedness modulates resting state neural activity in cortical midline structures. *Neuroscience*, *157*(1), 120–131.

Sugimura, K., Iwasa, Y., Kobayashi, R., Honda, T., Hashimoto, J., Kashihara, S., Zhu, J., Yamamoto, K., Kawahara, T., Anno, M., Nakagawa, R., Hatano, K., & Nakao, T. (2021). Association between long-range temporal correlations in intrinsic EEG activity and subjective sense of identity. *Scientific Reports*, *11*(1), 422. https://doi.org/10.1038/s41598-020-79444-2

Sui, J., Chechlacz, M., Rotshtein, P., & Humphreys, G. W. (2015). Lesion-Symptom Mapping of Self-Prioritization in Explicit Face Categorization: Distinguishing Hypo- and Hyper-Self-Biases. *Cerebral Cortex*, *25*(2), 374–383. https://doi.org/10.1093/cercor/bht233

Sui, J., He, X., Golubickis, M., Svensson, S. L., & Neil Macrae, C. (2023). Electrophysiological correlates of self-prioritization. *Consciousness and Cognition*, *108*, 103475. https://doi.org/10.1016/j.concog.2023.103475

Sui, J., Hong, Y., Hong Liu, C., Humphreys, G. W., & Han, S. (2013). Dynamic cultural modulation of neural responses to one’s own and friend’s faces. *Social Cognitive and Affective Neuroscience*, *8*(3), 326–332. https://doi.org/10.1093/scan/nss001

Sui, J., Liu, C. H., & Han, S. (2009). Cultural difference in neural mechanisms of self-recognition. *Social Neuroscience*, *4*(5), 402–411. https://doi.org/10.1080/17470910802674825

Sui, J., Rotshtein, P., & Humphreys, G. W. (2013). Coupling social attention to the self forms a network for personal significance. *Proceedings of the National Academy of Sciences*, *110*(19), 7607–7612. https://doi.org/10.1073/pnas.1221862110

Sui, J., Zhu, Y., & Han, S. (2006). Self-face recognition in attended and unattended conditions: An event-related brain potential study. *Neuroreport*, *17*(4), 423–427.

Tacikowski, P., & Nowicka, A. (2010). Allocation of attention to self-name and self-face: An ERP study. *Biological Psychology*, *84*(2), 318–324. https://doi.org/10.1016/j.biopsycho.2010.03.009

Tan, K. M., Daitch, A. L., Pinheiro-Chagas, P., Fox, K. C. R., Parvizi, J., & Lieberman, M. D. (2022). Electrocorticographic evidence of a common neurocognitive sequence for mentalizing about the self and others. *Nature Communications*, *13*(1), 1919. https://doi.org/10.1038/s41467-022-29510-2

Truong, G., Roberts, K. H., & Todd, R. M. (2017). I saw mine first: A prior-entry effect for newly acquired ownership. *Journal of Experimental Psychology: Human Perception and Performance*, *43*(1), 192–205. https://doi.org/10.1037/xhp0000295

van der Meer, L., Costafreda, S., Aleman, A., & David, A. S. (2010). Self-reflection and the brain: A theoretical review and meta-analysis of neuroimaging studies with implications for schizophrenia. *Neuroscience & Biobehavioral Reviews*, *34*(6), 935–946.

Whitfield-Gabrieli, S., Moran, J. M., Nieto-Castañón, A., Triantafyllou, C., Saxe, R., & Gabrieli, J. D. (2011). Associations and dissociations between default and self-reference networks in the human brain. *Neuroimage*, *55*(1), 225–232.

Wolff, A., Berberian, N., Golesorkhi, M., Gomez-Pilar, J., Zilio, F., & Northoff, G. (2022). Intrinsic neural timescales: Temporal integration and segregation. *Trends in Cognitive Sciences*, *26*(2), 159–173. https://doi.org/10.1016/j.tics.2021.11.007

Wolff, A., Di Giovanni, D. A., Gómez‐Pilar, J., Nakao, T., Huang, Z., Longtin, A., & Northoff, G. (2019). The temporal signature of self: Temporal measures of resting‐state EEG predict self‐consciousness. *Human Brain Mapping*, *40*(3), 789–803. https://doi.org/10.1002/hbm.24412

Woźniak, M., Kourtis, D., & Knoblich, G. (2018). Prioritization of arbitrary faces associated to self: An EEG study. *PLoS One*, *13*(1), e0190679.

Zhan, Y., Xiao, X., Tan, Q., Li, J., Fan, W., Chen, J., & Zhong, Y. (2020). Neural correlations of the influence of self-relevance on moral decision-making involving a trade-off between harm and reward. *Psychophysiology*, *57*(9), e13590. https://doi.org/10.1111/psyp.13590

Żochowska, A., & Nowicka, A. (2024). Subjectively salient faces differ from emotional faces: ERP evidence. *Scientific Reports*, *14*(1), 3634. https://doi.org/10.1038/s41598-024-54215-5
